# Forest stand characteristics drive the macronutrient composition of *Vaccinium* winter forage for cervids

**DOI:** 10.1002/eap.70182

**Published:** 2026-02-05

**Authors:** Annika M. Felton, Laura Juvany, Per‐Ola Hedwall, Adam Felton, Julia Erbrech, Alina Sayn, Julien Morel, Märtha Wallgren, Anders Jarnemo, Leonie Schönbeck, Robert Spitzer

**Affiliations:** ^1^ Southern Swedish Forest Research Centre Swedish University of Agricultural Sciences (SLU) Lomma Sweden; ^2^ AgroParisTech Palaiseau France; ^3^ INRAE, UR EFNO Nogent‐sur‐Vernisson France; ^4^ Department of Crop Production Ecology Swedish University of Agricultural Sciences (SLU) Umeå Sweden; ^5^ Food Security Unit Joint Research Centre (JRC) Ispra Italy; ^6^ Forestry Research Institute of Sweden Uppsala Science Park Uppsala Sweden; ^7^ Department of Wildlife, Fish and Environmental Studies Swedish University of Agricultural Sciences (SLU) Umeå Sweden; ^8^ School of Business, Innovation and Sustainability Halmstad University Halmstad Sweden

**Keywords:** conifer stands, forest density, forest management, moose, nutritional balancing

## Abstract

Shrubs of the genus *Vaccinium* serve as foundation species in boreal ecosystems as they define much of the structure of the ground vegetation and play key roles in many ecosystem services and processes. For example, *Vaccinium myrtillus* (bilberry) and *Vaccinium vitis‐idaea* (cowberry) constitute staple foods for several species of large herbivores (Cervidae, deer) in Northern Europe. However, changes to the tree layer from forestry practices have resulted in declines in habitat suitability and the abundance of these shrubs over recent decades. Here, we assess whether related changes to tree basal area and species composition also affect the macronutrient composition of these shrubs, and if so, how such alterations may influence food selection by moose (*Alces alces*). We sampled bilberry and cowberry twigs during wintertime in five study areas dispersed latitudinally in Sweden, using 65 forest stands dominated by Scots pine (*Pinus sylvestris*) or Norway spruce (*Picea abies*) that varied in age and site fertility, while also taking into account soil C:N, pH, and moisture. We found that the macronutrient composition of bilberry and cowberry forage was significantly altered by forest density and tree species composition. In denser and more spruce‐dominated forests (i.e., lower understory light), forage contained less nonstructural carbohydrates, but more protein and lignin, compared to shrubs growing in more open and pine‐dominated forests. We also found that the forage available in such shaded environments was closer to the presumed nutritional target balance of moose. Our results illustrate that management decisions influence the macronutrient composition of understory shrubs in a way that may be important for herbivore foraging choices. We suggest that a larger variation in forest structure, both within and among stands across the landscape, will provide cervids with greater variation in forage qualities, since even small differences in forest structure can increase the nutritional variation of the forage. We discuss our results in the context of plant resource allocation, herbivore nutritional balancing and game and forest management.

## INTRODUCTION

The understory vegetation represents a major proportion of plant diversity in northern forest ecosystems and plays important roles in ecosystem processes through its effects on litter decomposition, nutrient cycling, soil processes, and forest succession (Nilsson & Wardle, 2005; Sardans & Peñuelas, 2012). Moreover, the forest understory provides important habitat and food resources to a wide variety of forest‐dwelling wildlife. In boreal forests, shrubs of the genus *Vaccinium* (family Ericaceae) in particular are a widespread, structurally defining, and dominant component in large parts of the understory (Økland, 1996) and are thus considered foundation species (sensu Ellison, 2019). *Vaccinium myrtillus* (bilberry) and *Vaccinium vitis‐idaea* (cowberry) are clonal shrubs that propagate through horizontal rhizomes. Each plant individual normally consists of many ramets (Ritchie, 1955, 1956), which can live for several decades (Flower Ellis, 1971). Bilberry is deciduous and its aerial shoots reach 10–60 cm in height (Ritchie, 1956), whereas cowberry is evergreen and is limited to 10–30 cm in height (Ritchie, 1955).

These *Vaccinium* shrubs play key roles in many forest ecosystem services and processes, including carbon sequestration (Hensgens et al., 2020), shelter for other plants (Svensson et al., 1994), and the provision of food resources for vertebrates and invertebrates (Hanula et al., 2015; Hertel et al., 2018; Selås, 2001), as well as for human consumption and recreation (Hörnsten, 2000; Vaara et al., 2013). Of notable importance is the role that these shrubs play in the diets of the large herbivore communities of Northern Europe. Here, the twigs and leaves of bilberry and cowberry are staple food sources for deer species (cervids), including moose *Alces alces* (Cederlund et al., 1980; Wam et al., 2010), roe deer *Capreolus capreolus* (Barančeková et al., 2010), red deer *Cervus elaphus* (Krojerová‐Prokešová et al., 2010), and the introduced fallow deer *Dama dama* (Obidziński et al., 2013). For example, in Sweden, *Vaccinium* shrubs, including both bilberry and cowberry, may represent 25–50% of the diets of the aforementioned cervid species (%DNA, annual mean, Spitzer, 2019; % dry matter, winter, Felton, Holmström, et al., 2020).

Notably, bilberry and cowberry have decreased over recent decades in Fennoscandian forests (Hedwall et al., 2013; Hedwall & Brunet, 2016; Miina et al., 2009). For example, in the hemiboreal region of Sweden, ericaceous shrubs have experienced a 50% decrease in cover over the last half century (Hedwall, Gustafsson, et al., 2019). In addition to nitrogen deposition which decreases their competitive ability (Bobbink et al., 2010), and the short‐term negative impact of clear‐cutting on their growth (Atlegrim & Sjöberg, 1996), the major cause of the observed decline is the large‐scale loss of suitable understory habitat due to increasing forest density and changes to tree species composition as a consequence of forest management strategies (Hedwall, Gustafsson, et al., 2019). During the period of the observed decline, industrial‐scale efforts to boost wood production have increased standing timber volumes per hectare at final felling on more than 85% of the productive (producing ≥1 m^3^ of wood ha^−1^ year^−1^) forest land available for timber production (Felton, Löfroth, et al., 2020). Almost 80% of this standing volume is provided by just two native evergreen conifers, Norway spruce (*Picea abies*, hereafter spruce) and Scots pine (*Pinus sylvestris*, hereafter pine) (SLU, 2023). Spruce has more than double the leaf area index of pine (Felton, Petersson, et al., 2020) and a much greater negative impact on the understory light availability (Verheyen et al., 2012). The increased density of production forests, and the widespread use of these two conifers, has in turn reduced understory light levels in managed forests (Petersson et al., 2019), and thereby reduced the abundance of shade‐intolerant plant species (Hedwall, Gustafsson, et al., 2019), including *Vaccinium* shrubs (Hedwall et al., 2013; Hedwall & Brunet, 2016; Petersson et al., 2019). As darker stand conditions reduce the ground cover of these shrubs, their loss also reduces forage availability for cervids (Juvany et al., 2023). For example, in conifer stands with a basal area of 24 m^2^ ha^−1^, bilberry shrubs growing under a spruce‐dominated canopy (100% of total basal area) cover on average 32% less of the ground (Hedwall et al., 2025) and produce 29% less biomass per ramet annually than shrubs growing under a pine‐dominated canopy (Juvany, 2023).

What remains unknown is whether changes in the tree canopy layer of Fennoscandian forests are also influencing the macronutrient composition (Box 1) of *Vaccinium* shrubs, potentially causing additional compounding or compensatory effects on their consumers beyond the well‐documented shifts in their abundance. Both resource quantity and quality drive organism performances, and it is important to consider both aspects when assessing the impact of human land use on wildlife (Danger et al., 2022). Predictions regarding tree canopy effects on the understory plants can be made based on basic premises in plant ecology about how plants have evolved to allocate their resources. Plants growing under low light conditions are generally source limited; hence, carbon assimilation is limited directly by suppression of the photosynthetic system (Fatichi et al., 2014). In the case of sufficient availability of other resources, such as nitrogen, this would lead to reduced carbohydrate content and a relative increase of nitrogen‐based compounds. This includes, however, allocation of nitrogen to pigment–protein complexes to increase low‐light photosynthetic rates, in turn reducing the availability of nitrogen for other soluble proteins (Evans & Poorter, 2001). In most other cases, plants are generally assumed to be sink‐limited, meaning that stress from drought, nutrients, or temperature affects growth (carbon sink) more than photosynthesis (carbon source) (Körner, 2015). Thus, when plants have access to sufficient light but insufficient soil nutrients, growth declines more than photosynthesis (sink limitation), resulting in more carbohydrate accumulation relative to nitrogen (Bryant et al., 1983; Körner, 2015). While these shifts in carbon and nitrogen balance are still not fully understood (Gessler & Zweifel, 2024; Trugman & Anderegg, 2025), they are likely to be relevant to cervid foraging decisions, as indicated by recent findings that the balance between protein and non‐protein macronutrients (carbohydrates and fats) in forage is a strong determinant of moose diet choice during winter (Felton, Wam, et al., 2021; Spitzer et al., 2023).

BOX 1 **Background to nutritional balancing with focus on macronutrients**

**FIGURE 1:**
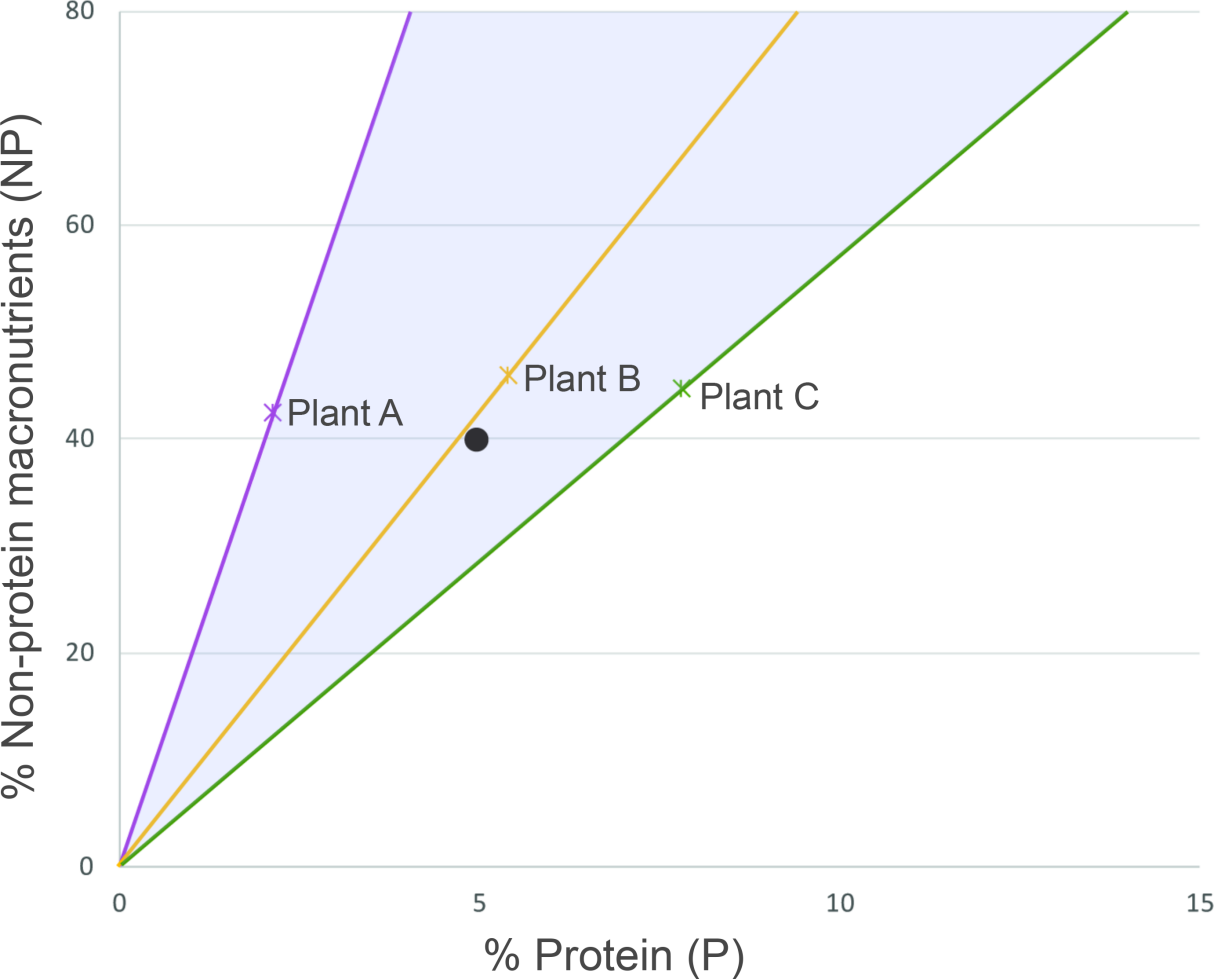
Illustration of how the balance between protein (P) and non‐protein macronutrients (NP) in tissues of forage items (plants A–C) can differ (P and NP can be expressed in energy or biomass units). The radials are called *food rails* and represent the nutritional balance of each item (*x* = laboratory value). The more an animal ingests (e.g., per meal or day), the further away from origin it ends up. When an animal is given uninhibited access to all food items, they can reach any point within the available *nutrient space* (shaded). The more items vary, the larger the nutrient space. Intake during uninhibited access indicates their *preferred target* (dot). While some forage items may provide a straight path to the target (Plant B), other items (A and C) may be combined to reach a similar composition. Such forage items are *complementary* (Simpson & Raubenheimer, 2012).

Previously, research into what governs herbivore diet selection often focused on 1–2 nutritional parameters in isolation (usually energy or protein; e.g., Belovsky, 1984). However, it is becoming increasingly clear that foraging should be seen as a dynamic process that involves balancing the intake of many different nutrients and anti‐nutrients to satisfy complex nutritional needs that change over multiple time scales (the nutrient balancing hypothesis, Simpson & Raubenheimer, 2012). Here, we focus on macronutrients, that is, energy‐providing compounds that occur in large amounts in plants. For ruminants, these include protein, non‐structural carbohydrates (sugars and starches), digestible structural carbohydrates (cellulose and hemicellulose), and fats. The nutritional state of an individual can change with each meal. Thus, an ideal food choice at one moment may be less suited at another. Animals deal with this complexity by selecting foods to achieve a *target nutrient balance* over a given time period, a finding replicated both in controlled experiments (e.g., Hewson‐Hughes et al., 2011) and in the wild (e.g., Raubenheimer et al., 2015). Animals continuously regulate the amounts they eat of different foods. While doing so, trade‐offs are unavoidable, as different nutrients come as food packages, not individual units. Many studies of nutritional balancing use the geometric framework for nutrition (Figure 1; Simpson & Raubenheimer, 2012).

Here, we assess the effect of stand characteristics on the macronutrient composition of bilberry and cowberry forage in Swedish conifer‐dominated production stands, focusing on the winter season when *Vaccinium* shrubs constitute a staple food for Swedish cervids in areas of low snow depth. The stand and site characteristics we assessed included tree species composition, forest density, time since stand‐replacing disturbance, and soil conditions. Because resource quality should be understood as a multidimensional property (Danger et al., 2022), in our assessment of forage quality we included all macronutrients (protein, fats, and [for ruminants] digestible carbohydrates; Box 1) as well as crude minerals (ash) and lignin. Lignin is a structural indigestible carbohydrate with functions related to both growth and defense of the plant. Compared to other defense compounds (such as phenols and tannins; e.g., Wam et al., 2018, not included in this study), lignin is a major component of woody plants (Moura et al., 2010).

We expected differences in the nutritional composition of *Vaccinium* forage present in pine versus spruce‐dominated forests, due to associated differences in understory light availability. Increasing forest density, here estimated by basal area, reduces light availability in both forest types, but this effect is more pronounced in spruce‐dominated stands (Hedwall et al., 2025). We acknowledge that the overstory influences the understory vegetation in multiple ways (Balandier et al., 2022), but we focus on light availability, as this is a major driver of photosynthesis and plant allocation of carbon and nutrients. We used tree basal area and the percentage of spruce as proxies for light availability, as these two variables combined have proven excellent predictors of canopy openness in our types of managed forests (Korhonen et al., 2007). Given the ecological relationships underlying plant resource allocation (see above), we hypothesized (H1) that bilberry and cowberry shrubs found in stands with high tree basal area/high percentage spruce and high site fertility should produce forage with relatively high protein (nitrogen) but low carbohydrate content compared to shrubs found in more open forest stands with lower site fertility. We place our findings in the context of cervid diet choice and nutritional balancing (Box 1), and for this, we used moose, a model species in nutritional ecology (Belovsky, 1984). Research into moose nutritional ecology has shown that while some highly preferred forage items lead moose on a straight path to their macronutrient target during winter time (e.g., willow, *Salix* spp.; Felton et al., 2016), a mixed intake of twigs from Scots pine and *Vaccinium* shrubs results in a similar nutritional composition (Spitzer et al., 2023). Our second hypothesis (H2) was therefore that the resultant changes in macro‐nutritional balance due to stand characteristics will affect how close the *Vaccinium* forage is to the moose's target balance between protein and non‐protein macronutrients (see Box 1, Figure 1), and we discuss the potential implications of these results for the shrubs' vulnerability to browsing.

## MATERIALS AND METHODS

### Study areas and study design

**FIGURE 2:**
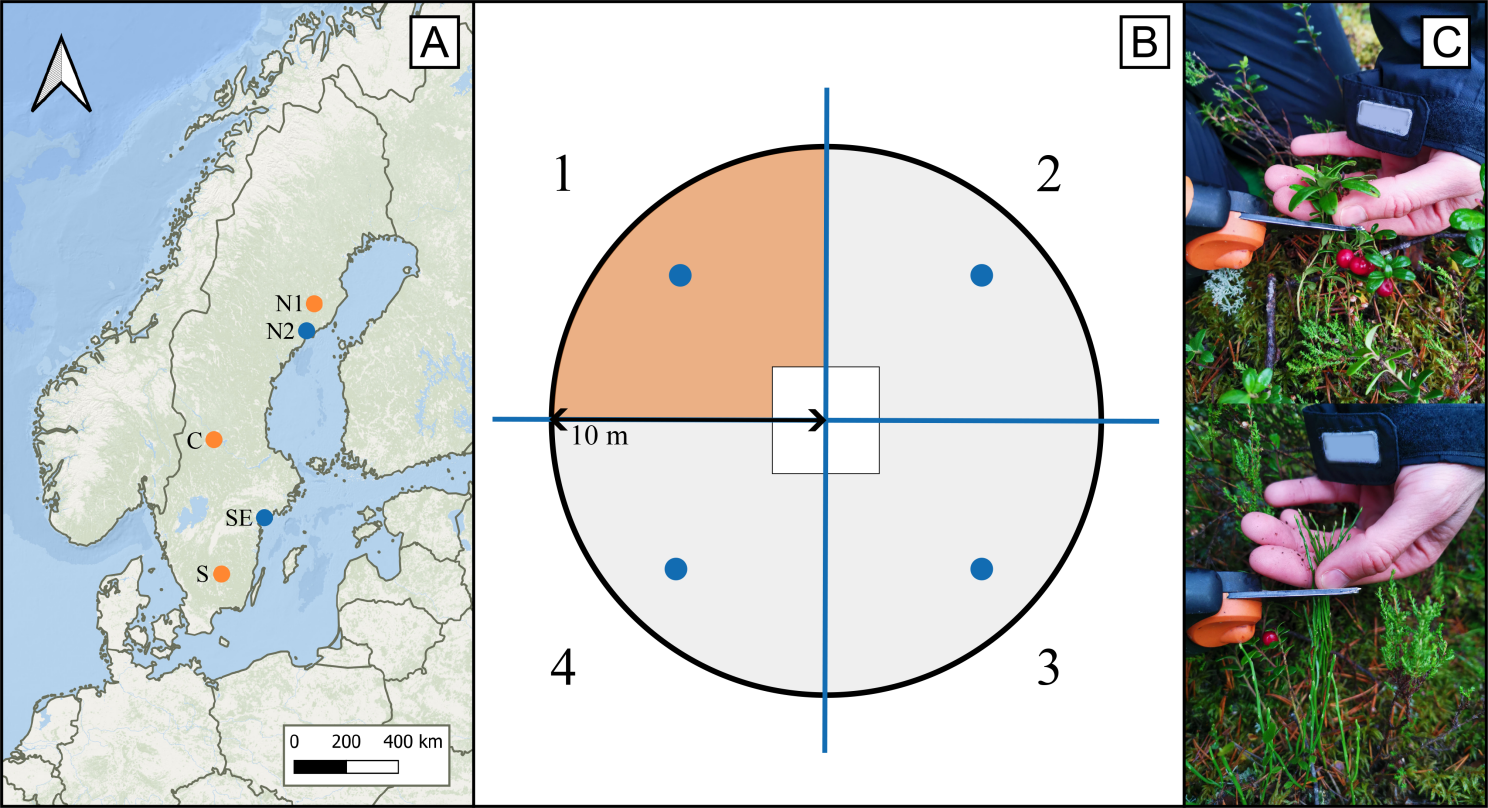
(A) Map showing the five study areas (dots), located in the north (N1 and N2), central (C), southeast (SE) and south (S) of Sweden. In total, 65 forest stands were sampled: 16 stands in area C, 15 in N1, 14 in S, and 10 in each of areas N2 and SE. (B) In each stand, bilberry and cowberry were sampled in two circular plots (*r* = 10 m in areas indicated with orange dots on the map; *r* = 8 m in areas with blue dots), avoiding a center square of 2 × 2 m (white box). Each plot was divided into four quadrants (1–4), of which one was randomly selected for the sample collection (orange shade). One soil sample was taken in each quadrant (blue dots). (C) Cowberry (above) and bilberry (below) were sampled by cutting small clusters of twigs of 3–4 cm in length from the top layer of the plants. Out of the 130 plots sampled, we found bilberry in 125 and cowberry in 123. Photos in panel (C) by Laura Juvany.

Forests cover 70% of Sweden's land area, spanning both temperate and boreal biomes. The majority of productive forest area is used for forestry, with most production forests managed using intensive even‐aged approaches that generally involve soil scarification, the planting of improved seedlings, repeated removal of competing vegetation, and finally clear felling at a fraction of the production trees' potential lifespans (Felton, Löfroth, et al., 2020). These forests are of course also subject to natural disturbances, such as storms, pathogens, and insect outbreaks, that also result in canopy gaps (Berglund & Kuuluvainen, 2021; Kuuluvainen & Aakala, 2011), but the overriding disturbance dynamics in Sweden's production forests are defined by anthropogenic inputs rather than natural disturbance processes. Likewise, forest fires are largely suppressed by humans. Our five study areas (N1, N2, C, SE, and S) contained forests comprised of even‐aged pine‐ or spruce‐dominated stands, which represent the vast majority of the country's forest area and also characterize the majority of habitats found in cervid home ranges. The study areas were located in lowland regions along a latitudinal gradient in Sweden (Figure 2A), covering a wide range of environmental and climatic variation (Table 1). Two study areas were located within the northern boreal forest region, with N2 having a stronger coastal influence than N1. C is located at the limit between the boreal and temperate regions, while SE and S, the two southernmost sites, are located within the temperate region.

**TABLE 1:** Climate data from the closest weather station (name given in brackets) for each study area.

Study area	Elevation (m)	Mean annual temp. (°C)	Annual snow cover (days)	Mean annual rainfall (mm)	Length of the vegetation period (days)	Sampling date
N1 (Vindeln‐Sunnansjönäs)	237	3.0	140–160	634.9	156	11–16 Oct
N2 (Nordmaling)	82	4.1	120–140	714.3	163	17–19 Oct
C (Siljansfors)	239	4.4	120–140	707.0	171	21–24 Oct
SE (Strängstorp)	48	6.8	40–60	627.3	199	11–15 Nov
S (Berg)	250	6.7	60–80	731.2	199	16–18 Nov

*Note*: Values are averaged for the reference period 1991–2020 and 1991–2013 for the annual snow cover. All data were obtained from the Swedish Meteorological and Hydrological Institute (SMHI, 2023).

We sampled in 65 forest stands, distributed so that study areas had between 10 and 16 stands (Figure 2A). For the areas N1, C, and S, stand selection was carried out using data available from research field stations located in each area, and was based on three stand characteristics: tree species composition, stand age, and site fertility. For tree species composition, stands were dominated (≥70% basal area) by either pine or spruce (Appendix S2: Table S1). Stands were selected to represent four age categories: 6–15, 16–48, 49–75 years, and ≥76 years. For each age category and tree species, we selected stands with contrasting site fertility estimates (low/high), using available data on site index (SI) from each study area (Appendix S1). Areas N2 and SE were opportunistically added to our research design (from another research project) and provided additional data for pine‐dominated stands of 49–75 years old. Across all five areas, data were collected in two circular plots per stand (radius either 8 or 10 m, with an area of 201 and 314 m^2^ respectively, Figure 2B). The plots were divided into four equal‐sized quadrants, of which one quadrant was randomly chosen for collecting samples during the winter of 2021.

### Sample collection

Moose and other deer in Sweden eat large amounts of *Vaccinium* during the cold months of the year (Spitzer, 2019), when the plants are dormant. Field sampling was therefore carried out after dormancy was expected to have set in, which for bilberry buds is known to be after a 10‐day period with air temperatures not exceeding 11°C (Jenderek et al., 2017). Due to the large latitudinal gradient in our study, this criterion was met at different dates in our study areas (SMHI, 2025). Thus, field sampling was carried out from 11 October to 18 November 2021, starting in the north and continuing southwards. We assume that the plants' nutritional composition at the time of collection is roughly representative for the majority of the winter period, while acknowledging that some plant metabolic activity occurs during dormancy, especially during mild winters (Perry, 1971). In each plot (total 130), we collected samples from either one or both species, depending on their presence. As bilberry is a deciduous species, the twigs we collected lacked leaves. In contrast, samples of the evergreen cowberry included not just twigs but also leaves. We cut small clusters of twigs of 3–4 cm in length from the top layer of the plants (Figure 2C) to mimic the feeding behavior of cervids (as per Felton, Wam, et al., 2021; Spitzer et al., 2023). We refer to this method as the “mouthful” method, and the collected material equates to “forage.” We collected material until we had reached 60–100 g wet mass per plant species and plot. Samples were collected starting from close to the center of the plot to maximize the relevance of tree layer measurements (see below) to understory responses. However, samples were not taken at the center of the plot (Figure 2), as this area consisted of a 2 × 2 m square used in a parallel research project. Samples were stored at −20°C shortly after collection.

### Plot measurements and calculations: Tree layer, site index, and soil samples

Tree species and dbh (1.3 m from the ground) of all present living trees taller than 1.3 m were recorded within each plot (Figure 2). We calculated the total basal area per plot (in square meters per hectare) as well as each tree species' contribution to it. We estimated stand age by coring trees and calculated our own value of stand site index (SI_*c*_; tree height in meters at 100 years' age; Appendix S1). Within each circular plot, but outside the 2 × 2 m center square, we collected a total of four soil samples (Figure 2; Appendix S1). The four samples from the same plot were pooled together. In brief, the total carbon (C) and nitrogen (N) content in the soil was determined (Appendix S1), and the C:N ratio was calculated. Nitrogen is the main nutrient that restricts productivity in our study systems, and the C:N ratio is a useful proxy for variation in soil nitrogen availability (Hedwall, Holmström, et al., 2019). We also measured soil pH (Appendix S1) and extracted a soil moisture value for the center of each plot through the SLU moisture map (SLU Markfuktighetskarta; Ågren & Lidberg, 2020), which has a 2 × 2 m resolution.

### Sample preparation and chemical analysis

We obtained 125 samples of bilberry and 123 samples of cowberry. Samples were weighed before drying at 60°C until they came to a constant mass (normally 24 h) and then ground using a cutting mill (Retsch SM 2000 and Retsch SM 300; 1 mm sieve). Due to the high costs associated with wet chemistry analyses, we used near‐infrared spectroscopy (NIRS) to estimate concentrations of nine nutritional constituents (see below), with a subset of representative samples also analyzed using wet chemistry for calibration purposes (as per Vance et al., 2016). NIRS reflectance spectra were acquired with a hyperspectral camera (Appendix S1). Following data scanning, we selected a representative subset of samples using distance‐based sampling within the principal components analysis space. This approach ensured that the selected samples spanned the range of variation in nutritional constituent concentrations observed in the complete dataset. Distances were calculated using the first two principal components, which collectively explained 95%–98% of the total variance. Specifically, 43 bilberry and 44 cowberry samples were selected (i.e., 35% of the whole population of samples) and sent to the laboratory DairyOne©, USA, for chemical analyses. Plant samples were analyzed for the constituent ash, total nitrogen, acid‐detergent fiber (ADF), ADF‐N (insoluble nitrogen within the ADF fraction), crude fat, amylase and sodium sulfite‐treated neutral‐detergent fiber (aNDF), lignin, starch and water‐soluble carbohydrates (WSC), using conventional wet chemistry techniques (Appendix S1). Results from the wet chemistry analyses of the 87 representative plant samples were used to adjust multivariate regression models where NIRS spectra were used as explanatory variables and above‐mentioned laboratory‐measured traits were used as response variables (Appendix S2 Table S2).

We calculated available protein (AP) as total protein (total N × 6.25) minus non‐digestible protein (ADF‐N × 6.25) (Licitra et al., 1996). Hemicellulose was calculated as aNDF–ADF and cellulose as ADF–lignin. In addition to being necessary for the calculation of cellulose, information about lignin is valuable in itself. Although ruminants cannot obtain energy from this structural carbohydrate, it is part of the forage roughage that sustains a relatively stable environment in the rumen (Allen, 1997). Lignin also functions as a barrier that protects the plant against pests and pathogens (Liu et al., 2018), and as a digestion inhibitor for ruminants (Van Soest, 1994). We calculated the energetic value of each food using the following conversion factors (National Research Council, 1989): 37.7 kJ g^−1^ lipid and 16.7 kJ g^−1^ AP and total non‐structural carbohydrates (TNC = sum of starch and WSC), cellulose, and hemicellulose.

TNC, cellulose, and hemicellulose were summed to obtain total carbohydrates (TCH), and for each sample, we calculated the AP:TCH ratio, which has been shown to be useful when relating forage nutritional composition in relation to the target balance (Box 1) of moose. Note that lignin, which for cervids is an indigestible carbohydrate, is not included in TCH. For comparison with our bilberry and cowberry data, we used published data on the AP:TCH ratio of dormant forage of pine (twigs with needles) and *Salix* spp. (twigs without leaves) from Spitzer et al. (2023, collected in a large variety of habitat types within study areas N2 and SE) and Felton, Wam, et al. (2021, collected in unfertilized young pine stands in south‐central Sweden). The same “mouthful” collection method and the same sample preparation and chemical assay procedures were used in all three studies (i.e., this study; Felton, Wam, et al., 2021; Spitzer et al., 2023), thereby avoiding erroneous comparisons (Zaguri et al., 2022). Note that in studies of nutritional balancing, one often uses the ratio protein (P): non‐protein macronutrients (NP), where NP not only includes digestible carbohydrates but also fats (Box 1). Due to potentially erroneous fat estimations, fat was excluded from this sum by Spitzer et al. (2023). To be able to compare, we did the same in the present study.

### Data analysis

We used R version 4.2.3 (R Core Team, 2023) for all statistical analyses. We applied mixed‐effects models to account for the hierarchical structure of our data, which were collected in plots that were geographically aggregated in stands, which in turn were aggregated in study areas. We carried out linear mixed‐effects models (LMM) to assess variations in concentrations of the measured chemical variables (AP, fat, cellulose, hemicellulose, lignin, TNC, and energy) between the two plant species, as these variables approximated a normal error distribution and error homoskedasticity. Stand and study area were included as nested random intercept variables following our design.

Two steps were needed to test the first hypothesis (H1, regarding the influence of stand structure on macronutrient composition of the forage). Due to our a priori interest in macro‐nutritional balancing by cervids (Box 1), we used a combined measure of the macronutrient composition in each plant sample rather than treating each nutritional variable in isolation. The first step was therefore to quantify the composition per sample by using one principal components analysis (PCA) per plant species, using the function PCA in the *FactoMineR* package (Lê et al., 2008). The macronutrients included in the PCA were AP, TNC, cellulose, hemicellulose, and crude fat. In addition, we included ash (crude minerals) and lignin.

As a second step, we used scores on principal components 1 and 2 from the PCA to test relationships between their nutritional composition and stand characteristics, using LMM as described above. Due to the large differences in the range of our predictors, we centered and standardized all independent variables, subtracting their mean and dividing by their SD. After carrying out a collinearity test for all independent variables, we found mean stand age and total basal area to be strongly positively correlated (Spearman correlation coefficient >0.6), which is why we included basal area as an indicator of light in our models. Spruce and pine percentage of the total basal area in a plot were negatively correlated with each other (due to our selection of stands dominated by either pine or spruce). Because the percentage broadleaves (predominantly birch, *Betula* spp.) in these forest stands was low (mean 2.6% of total plot basal area), we assumed that this variable did not influence the understory to a large extent. Therefore, after taking the above issues into consideration (variable collinearity and relevance), the stand characteristics that we included in the models were: total plot basal area (all tree species summed), the percentage of this basal area that was composed of spruce trees (hereafter “% spruce”) and the interaction between basal area and % spruce, as well as soil C:N, soil pH, soil moisture, and site index. The interaction between basal area and % spruce was included, as these variables in combination are demonstrated to be a good proxy for tree canopy openness (Korhonen et al., 2007), as well as the abundance of bilberry and cowberry (Hedwall et al., 2025). Study area and stand were included as nested random intercept variables following our design. Five outliers for bilberry and four for cowberry were removed in connection with analyses that concerned stand characteristics (due to presumed measurement errors regarding C:N ratio and stand index, see Appendix S1). The resultant sample size was therefore 121 for bilberry and 119 for cowberry.

Generalized linear mixed models with a beta error distribution (logit‐link function) were used to test the second hypothesis (H2, regarding the nutritional balance of the forage in relation to stand characteristics). The beta distribution was found to be suitable as the response variable was a ratio bounded between zero and one. To be able to compare with published data on the macronutrient composition of pine and *Salix* spp. forage (see above), we used the ratio between AP and TCH (total carbohydrates) as a response variable. We tested the relationship between the AP:TCH ratio and the same stand characteristics as were included in the LMM model described above (as fixed effects). After a first exploration of our modeling results, we suspected % spruce to have a nonlinear effect on our dependent variables (AP:TCH for bilberry and cowberry). Hence, we added the second polynomial of % spruce (% spruce^2^) to model this. Also, the variables study area and stand were included as nested random intercept in the models. Due to an additional outlier identified for the AP:TCH for bilberry (presumed measurement error), its sample size was therefore 120 for this model.

LMM and GLMM, where we used PC1, PC2, and AP:TCH as response variables, were fitted by applying the glmmTMB function in the *glmmTMB* package (Brooks et al., 2017). Model coefficients of determination (conditional and marginal *R* ^2^), as developed by Nakagawa et al. (2017), were calculated using the function r2 in the *performance* package (Lüdecke et al., 2021). We first ran full models with all the stand characteristics listed above as fixed effects in our model, and then used the dredge function from the *MuMIn* package (Barton & Barton, 2015) to obtain models with all possible variable combinations and select the model with the lowest Akaike information criterion (AIC). Our evaluation of the selected best models, using residual plots and QQ‐plots based on simulated residuals from the simulateResiduals function in the *DHARMa* package (Hartig, 2024), did not reveal any discrepancies from the model assumptions.

To illustrate how bilberry and cowberry forage differ in their AP:TCH ratio depending on stand characteristics, and how this relates to the presumed nutritional target balance for moose (see above), we defined two scenarios. Both scenarios considered the tree species composition and basal area of the stand. In the first scenario, we explored the effects of basal area, and in the 2nd scenario, we kept basal area constant and instead explored the effect of tree species composition, in terms of the proportion of pine or spruce. Specifically, in these two scenarios: (1) Tree composition was fixed on 70% spruce but total plot basal area was either 20 or 40 m^2^ ha^−1^. We used these values because in spruce‐dominated forests, the optimal stand density for bilberry is expected to be around 20 m^2^ ha^−1^ (Eldegard et al., 2019), and doubling that value would be a suitable contrast while also representing a density range commonly found in Swedish pine and spruce production stands (Persson et al., 2022). (2) Tree composition was either 0%–30% spruce or 70%–100% spruce, while keeping total basal area fixed at the mean value for the study (23 m^2^ ha^−1^). The predicted values used in the scenarios were based on the output from the best models for each plant species (Appendix S1).

## RESULTS

### Relationship between stand characteristics and the macro‐nutritional balance of bilberry and cowberry (hypothesis 1)

**FIGURE 3:**
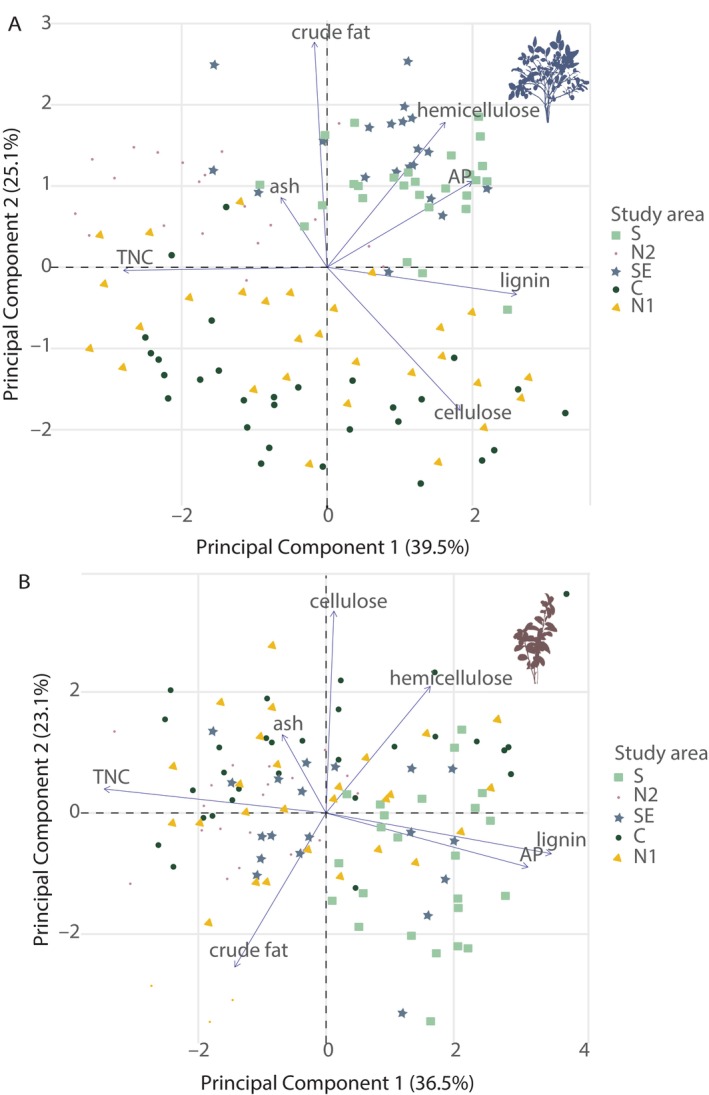
Bi‐plot combining loadings and scores from principal components analysis of the macronutrient composition of bilberry (A) and cowberry (B) forage samples collected during wintertime in Sweden. For each plant sample, the concentrations (in grams per 100 grams of dry matter) of available protein (AP), ash, crude fat, total non‐structural carbohydrates (TNC), cellulose, hemicellulose, and lignin are included in the model. Arrows indicate the correlation between a macronutrient parameter and the principal components (*x* and *y* axes; percentages refer to the variation explained by each principal component), and dots indicate positions of individual plant samples along the principal components, with the symbol and color specifying each of the five study areas (N1, N2, C, SE and S, Figure 1). Plant illustrations by Laura Juvany.

Concentrations of all seven macronutrient constituents differed significantly (*p* < 0.001 in all cases, Appendix S2: Table S3) between bilberry and cowberry forage. For example, cowberry forage (twigs and leaves) contained on average 69% more TNC (mean 27 g 100 g^−1^ ± 3.45 SE vs. 16 g 100 g^−1^ ± 2.1 SE), 43% more crude fat (5 g 100 g^−1^ ± 0.4 SE vs. 3.5 g 100 g^−1^ ± 0.6 SE), and 22% more energy (11 kJ g^−1^ ± 0.6 SE vs. 9 kJ g^−1^ ± 0.3 SE) than bilberry forage (twigs only). Both bilberry and cowberry exhibited large within‐species variation in the relative proportions of macronutrient variables (PCA, Figure 3, for component coefficients see Appendix S2: Table S4). For bilberry, 65% of the total variation was explained by the first two PCs, and for cowberry the equivalent value was 60%. For both species, PC1 was most strongly correlated with TNC in one direction, and with lignin, and to a varying extent also AP, in the opposite direction (Figure 3, Appendix S2: Table S4). Lignin and AP co‐varied more closely in cowberry than in bilberry. Crude fat and cellulose were negatively correlated with each other in both plant species, in almost opposite directions along PC2 (Figure 3). Hemicellulose and cellulose co‐varied in cowberry samples, but not in bilberry (Figure 3).

There was large variation among plots with regard to age and the tree basal area (Appendix S2: Table S1). With respect to soil conditions, plots varied as follows: soil C:N ranged from 19 to 47 (mean = 32), soil moisture from 0% to 88% (mean = 22%), and soil pH from 2.7 to 4.1 (mean = 3.2). The percentage of total basal area consisting of spruce within the plots ranged from 0% to 100%, with stands (mean of the two plots) ranging between 0%–25% and 53%–100% (Appendix S3: Figure S1). The best model according to AIC (marginal *R* ^2^ = 0.39, Table 2; full model in Appendix S2: Table S5) shows that scores on PC1 for bilberry samples were significantly and positively related to plot basal area (*p* < 0.001) and % spruce (*p* = 0.012). In other words, the higher the plot basal area, and the larger the proportion of that basal area made up of spruce, the higher the concentrations of protein (AP) and lignin in the bilberry samples. Additionally, soil C:N was negatively related to PC1 (*p* = 0.042). The interaction between plot basal area and % spruce was also included in the best model, despite not being significant (Table 2), because the positive coefficient of this interaction may indicate a trend towards a stronger effect of basal area at higher % spruce than at low. The best model for cowberry (marginal *R* ^2^ = 0.43, Table 2; full model in Appendix S2: Table S6) shows similar results with regard to scores on PC1, as these were significantly and positively related to plot basal area (*p* < 0.001) and % spruce (*p* < 0.001; Table 2). There was also a significant interaction between plot basal area and % spruce (*p* = 0.006). This indicates that for cowberry, the positive relationship between basal area and the value of PC1 (and thus available N and fiber) is stronger when the proportion of spruce is larger. We could not predict the values of bilberry along PC2 as the best model produced a marginal *R* ^2^ of only 0.016 (Table 2, Appendix S2: Table S5). For cowberry, the best model performed better (marginal *R* ^2^ = 0.19) and shows that scores on PC2 (which represented variation in crude fat and digestible structural carbohydrates) were significantly related to % spruce (positive, *p* = 0.002) and site index (negative, *p* = 0.013; Table 2, Appendix S2: Table S6).

**TABLE 2:** Linear mixed models (LMM) describing the relationship between stand characteristics (centered and standardized variables) and the principal components (PC1 and PC2) in Figure 3, which is a measure of the macro‐nutritional balance of the bilberry and cowberry forage.

Model	Factor	Total basal area	% spruce	Total basal area × % spruce	Soil C:N	Soil pH	Site index (SI_*c*_)
Bilberry							
PC1^a^	Coefficient	0.73	0.30	0.14	−0.27		
	SE	0.12	0.12	0.09	0.14		
	*z*‐statistic	6.07	2.50	1.54	−2.03		
	*p* value	**<0.001**	**0.012**	0.123	**0.042**		
PC2^b^	Coefficient	−0.13				0.13	
	SE	0.06				0.06	
	*z*‐statistic	−2.09				2.01	
	*p* value	**0.037**				**0.044**	
Cowberry							
PC1^c^	Coefficient	0.61	0.52	0.23	−0.21		
	SE	0.10	0.10	0.08	0.13		
	*z*‐statistic	5.87	4.94	2.77	−1.58		
	*p* value	**<0.001**	**<0.001**	**0.006**	0.115		
PC2^d^	Coefficient	0.22	0.36				−0.36
	SE	0.12	0.12				0.14
	*z*‐statistic	1.91	3.09				−2.47
	*p* value	0.056	**0.002**				**0.013**

*Note*: For each PC, the table shows the results from the best model per species, obtained through model selection from full models (see *Materials and methods*). For full model results and candidate models, see Appendix S2: Tables S5 and S6. Bold values indicate significant effects (*p* value < 0.05).

^a^ Marginal *R* ^2^ = 0.39; conditional *R* ^2^ = 0.80.

^b^ Marginal *R* ^2^ = 0.02; conditional *R* ^2^ = 0.86.

^c^ Marginal *R* ^2^ = 0.43; conditional *R* ^2^ = 0.65.

^d^ Marginal *R* ^2^ = 0.19; conditional *R* ^2^ = 0.52.

### Stand characteristics influence how close bilberry and cowberry forage are to the macro‐nutritional target balance for moose (hypothesis 2)

**FIGURE 4:**
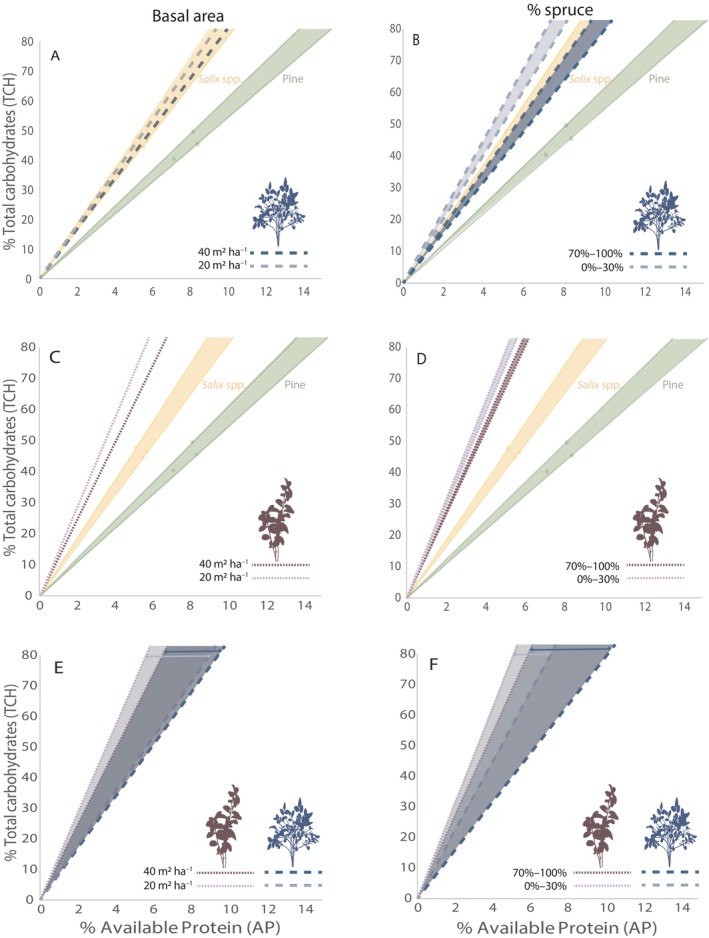
(A–D) Macronutrient composition in the dimensions of available protein (AP) and total carbohydrates (TCH, which includes cellulose, hemicellulose, and all non‐structural carbohydrates) in bilberry (twigs, blue dashed lines, panels A, B) and cowberry (twigs + leaves, purple dotted lines, panels C, D), in response to total plot basal area (A, C) and % spruce (B, D). Lighter colors indicate our lower values of basal area (20 m^2^ ha^−1^) and % spruce (0%–30%); darker colors represent higher basal area (40 m^2^ ha^−1^) and % spruce (70%–100%; see *Materials and methods*). The values are presented in comparison with the average composition of Scots pine (green dots and food rail; see Box 1 for terminology) and *Salix* spp. (yellow dots and food rail), as published in Felton, Wam, et al. (2021) and Spitzer et al. (2023). (E, F) Values of panels A–D are combined to indicate the differences between bilberry and cowberry (and the resulting nutritional space the two species create together (Box 1), the width of which is indicated by arrows) in response to variation in basal area and % spruce; light gray shade represents lower basal area and % spruce (as defined above) and dark gray shade indicates higher basal area and % spruce. Plant illustrations by Laura Juvany.

The best model according to AIC showed that AP:TCH (available protein:total digestible carbohydrates) in bilberry was significantly positively related to plot basal area (*p* = 0.004) and % spruce (*p* = 0.010; Table 3). Additionally, the quadratic effect of % spruce (% spruce^2^) had a significant negative effect in the bilberry model (Table 3). This indicates that AP:TCH follows a nonlinear response to increasing % spruce. Similar results were found for cowberry (plot basal area *p* < 0.001, % spruce *p* = 0.016; Table 3), except that no nonlinearity was indicated. Therefore, bilberry and cowberry shrubs with dense and spruce‐dominated overstories produced forage that was closer to the ratio found in *Salix* twigs, and by extension the presumed macro‐nutritional target balance for moose (Box 1; Felton et al., 2016; Spitzer et al., 2023). The bilberry forage in spruce‐dominated forests (70% spruce) even overlapped in composition with our reference values for *Salix* spp., regardless of variation in basal area (20 vs. 40 m^2^ ha^−1^; Figure 4A). Bilberry forage similarly overlapped with *Salix* spp. in forests with high % spruce (70%–100%), but not in forests with low % spruce (0%–30%; Figure 4B [plot basal area 23 m^2^ ha^−1^ in both cases]). The AP:TCH in cowberry forage differed less among stand types and was never as close to *Salix* spp. as bilberry was (Figure 4C,D). The nutritional space created by bilberry and cowberry together was larger in forests with high % spruce than low % spruce (Figure 4F), while the difference in nutritional space was similar regardless of variation in basal area, given it was a spruce‐dominated stand (Figure 4E).

**TABLE 3:** Generalized linear mixed models (GLMM) describing the relationship between stand characteristics (centered and standardized variables) and the ratio between available protein (AP) and total carbohydrates (TCH = TNC (sugar + starch) + cellulose + hemicellulose) in bilberry and cowberry forage.

Model	Factor	Intercept	Total basal area	% spruce	% spruce^2^	Soil C:N
Bilberry						
AP:TCH^a^	Coefficient	−2.30	0.04	0.15	−0.14	−0.06
	SE	0.04	0.01	0.06	0.06	0.02
	*z*‐statistic	−56.18	2.86	2.57	−2.41	−3.40
	*p* value	**<0.001**	**0.004**	**0.010**	**0.016**	**0.001**
Cowberry						
AP:TCH^b^	Coefficient	−2.65	0.10	0.06		−0.05
	SE	0.07	0.02	0.02		0.03
	*z*‐statistic	−39.75	4.78	2.41		−1.58
	*p* value	**<0.001**	**<0.001**	**0.016**		0.113

*Note*: For each plant species, the table shows results from the best model obtained through model selection from full models (see *Materials and methods*). For full model results and candidate models, see Appendix S2: Table S7. Bold values indicate significant effects (*p* value < 0.05).

Abbreviation: TNC, total non‐structural carbohydrates.

^a^ Marginal *R* ^2^ = 0.24; conditional *R* ^2^ = 0.76.

^b^ Marginal *R* ^2^ = 0.24; conditional *R* ^2^ = 0.60.

## DISCUSSION

Our key finding was that the stand characteristics in conifer production forests significantly influenced the macronutrient composition of the *Vaccinium* shrubs, providing support to our primary hypothesis. The driving factor behind cervid foraging is to obtain sufficient nutrients and energy to survive and grow (Felton et al., 2018), and to do so, they may ingest large amounts of *Vaccinium* shrubs, particularly during wintertime in areas where snow depth still allows access to the understory vegetation (Cederlund et al., 1980; Spitzer, 2019). Our study revealed that the decline observed over recent decades in the quantity of *Vaccinium* shrub forage for cervids in Sweden (Hedwall et al., 2013; Hedwall, Gustafsson, et al., 2019) is coupled with a significant modification of its nutritional composition. We start by discussing the likely physiological underpinnings of why the plants exhibited the observed response to differences in the canopy (H1) and then interpret these results in the context of nutrient balancing by cervids (H2) because the observed pattern has potential consequences for cervid diet choice and forest damage.

### Relationship between stand characteristics and the macronutrient composition of bilberry and cowberry (hypothesis 1)

Our results for both bilberry and cowberry showed that forest density and percentage of spruce were negatively related to the concentration of sugars and starches (TNC), but positively related to the concentration of AP (Table 2, Figure 3). This confirms the theory from a source versus sink limitation perspective, stating that light limitation leads to reduced carbohydrate concentrations in plants due to reduced photosynthesis (Fatichi et al., 2014; Körner, 2015). Support for this theory also comes from a meta‐analysis conducted by Koricheva et al. (1998) on 107 woody plant species showing that carbohydrate concentrations (TNC) in leaves were indeed significantly reduced by decreased light availability. As a result, and to increase photosynthetic capacity in the form of increased Rubisco content, plant tissues are expected to have higher concentrations of nitrogen (Evans & Poorter, 2001). This is reflected in our results regarding AP. A positive relationship between tissue nitrogen concentration and shade (or basal area) has been found in studies of other ericaceous shrubs (Michelsen et al., 1996; van Horne et al., 1988), and cervid forage plants, for example, oak trees *Quercus* spp. (Baraza et al., 2004; Estiarte et al., 2007). Another factor that contributed to a smaller degree to carbon–nitrogen dynamics in the plant tissues was soil quality. Although soil C:N featured in our best models for both plant species with regard to PC1, it was only significant for bilberry (*p* = 0.042; C:N was also present and often significant in other top candidate models for bilberry, Appendix S2: Table S5). When present in a model, soil C:N had a negative effect on PC1 and therefore AP. This was expected, as the larger the soil C:N, the less nitrogen is available for the plants. However, our model results suggest that the effects of the tree layer were larger than the effects of soil quality on the plants' resource allocation, at least with respect to the chemical composition of their winter‐dormant tissues. Accordingly, previous research has found that changes in the biomass (Mäkipää, 1999), photosynthetic physiology (Palmroth et al., 2014), and palatability (Schrijvers‐Gonlag et al., 2020) of these plant species are mostly associated with responses to the tree canopy rather than to soil nutrient conditions.

While our results regarding nonstructural carbohydrates (TNC) and nitrogen (AP) were in line with expectations, the results regarding the other macronutrients (cellulose, hemicellulose, and fat) are harder to explain. The plants' concentration of hemicellulose was not clearly related to either principal component, which meant we could not discern a relationship with stand characteristics. Although the variation in cellulose and fat was captured well by PC2, the models we used to test relationships with stand variables had fairly low marginal *R* ^2^ for both bilberry (0.02) and cowberry (0.19). The relatively large conditional *R* ^2^ in these models (0.52 and 0.86, respectively) indicated that most of the variation was explained by the random‐effect study area and local factors that we did not measure. Such factors could, for example, be temperature and day length, which are suggested to influence the dormancy process (Cuny & Rathgeber, 2016; Perry, 1971).

Our data were more definitive with respect to lignin concentrations in bilberry and cowberry being positively related to high basal area and % spruce (Table 2). The functional roles of lignin in woody plants include both mechanical support and defense (Moura et al., 2010). We do not think that differing needs for mechanical support by our study plants can explain the patterns observed. Bilberry ramets growing in open patches tend to be more upright, likely requiring more, not less, lignin than those growing in shade (Tolvanen, 1995). Lignin also functions as a digestion inhibitor for ruminants, which causes these herbivores to avoid ingesting large amounts of lignin‐rich biomass (Van Soest, 1994). In dense, spruce‐dominated forests, bilberry and cowberry ramets are significantly smaller than in pine‐dominated forests (Juvany et al., 2023), but a larger proportion of their biomass is represented by fresh annual shoots that cervids use as forage (Juvany, 2023). Coupled with shade‐induced higher levels of protein, this could make a larger part of the ramet attractive as forage, and—to add to their protection—shrubs may consequently increase lignin levels. The synthesis of lignin compounds has been found to be upregulated when herbivory defense response is induced in bilberry (through activation of the jasmonic acid pathway) (Benevenuto et al., 2019). However, a long‐term study of simulated browsing by clipping did not result in an increase in lignin concentrations in bilberry (Persson et al., 2012). The relationship between herbivory and lignin production in bilberry and cowberry therefore remains an open question. Similarly, the relationship between light intensity and lignin synthesis remains unclear. Lignin synthesis in most plants is stimulated by light (Hussain et al., 2019), which should result in reduced lignin synthesis in lower light conditions. However, exceptions occur, particularly among the gymnosperms. For example, Scots pine and Norway spruce increase their lignin synthesis in more shaded environments, likely as a defense mechanism and adaptation to northern growing conditions (Ranade et al., 2022a, 2022b). Notably, our study showed higher lignin concentrations under low light conditions in understory angiosperms, but the exact driver remains unclear.

### Stand characteristics influence the macro‐nutritional balance of bilberry and cowberry forage in relation to the target balance for moose (hypothesis 2)

In our study, the nutritional composition of bilberry and cowberry forage was closer to the macro‐nutritional rail of *Salix* spp. in forests with high basal area and high % spruce, compared to forests that were more open and dominated by Scots pine (Figure 4). By extension, the available shrub forage in dense forests was closer to the presumed macro‐nutritional target balance for moose (Felton et al., 2016; Spitzer et al., 2023). The hypothesis that twigs of willows (*Salix* spp.) possess a well‐balanced nutritional composition was first proposed by Felton et al. (2016), based on a feeding experiment with captive moose. This observation has since gained further support through studies of free‐ranging moose in both winter (Spitzer et al., 2023) and summer (Spitzer et al., 2024). Furthermore, moose populations in southern Sweden that include relatively large amounts of *Salix* forage in their winter diet have larger calf weights and higher reproductive rates than moose that do not (Felton, Holmström, et al., 2020).

Interestingly, we found that the AP:TCH of bilberry forage actually overlaps with the composition of *Salix* spp. when growing in spruce‐dominated stands, regardless of variation in basal area for the range used in our predictions (Figure 4A,B). Consumption of such forage should more directly lead moose to their macro‐nutritional target during winter, which in turn could have implications for the palatability of these shrubs. Notably, previous research has shown that insect herbivory on bilberry leaves increases with increasing shade (Schrijvers‐Gonlag et al., 2020). The forage of cowberry did not show this overlap with *Salix* spp. in any of the scenarios (Figure 4C,D), and there appear to be slight differences in how the two shrub species adjust to shade. This could be because cowberry is an evergreen species while bilberry is deciduous, which likely affects their light adaptation, as a high degree of plasticity in light response has been found among other *Vaccinium* shrubs that differ in this regard (Kawamura & Takeda, 2002). A food item does not, however, need to be perfectly on‐target to be of value to the consumer. As Figure 4 shows, *Vaccinium* shrubs are nutritionally complementary food items in relation to other common moose forage species, such as young Scots pine trees (Felton, Wam, et al., 2021; Spitzer et al., 2023), at least with respect to the parameters assessed in this study. In absolute terms, *Vaccinium* shrubs are fairly low in digestible protein and energy, even compared to other woody forage (Felton, Wam, et al., 2021). The availability of *Vaccinium* shrubs is therefore sometimes considered to be of less importance than the availability of moderate‐ to high‐energy forage when explaining trends in cervid fitness (e.g., moose, Schrempp et al., 2019). At the scale of an individual's diet choice, however, the conventional view of food items being of “high” versus “low” quality or palatability may need adjustment. This is because the value of a particular food to a feeding animal is not fixed, but changes with time and circumstance as the sought‐after food constituent(s) alter in response to each meal consumed (Felton et al., 2018; Raubenheimer et al., 2014; Simpson & Raubenheimer, 2012).

A larger variety of trees, shrubs, herbs and grasses provides the animals with a larger nutritional space to maneuver within (Felton, Wam, et al., 2021), which increases their ability to reach their macro‐nutritional target and to avoid high doses of secondary metabolites (Villalba & Provenza, 2005; Wam et al., 2018). Because *Vaccinium* shrubs represent a substantial proportion of cervid diets, any changes by forestry that modify the nutritional composition of this forage on a large spatial scale may alter the nutritional landscape these animals navigate within. Such changes to the nutritional landscape can impact individual fitness over time (Felton, Holmström, et al., 2020), and ultimately, population dynamics (Parker et al., 2009; Schrempp et al., 2019). Our results (Figure 4E,F) indicate that a mixture of different stand types in a landscape will provide cervids with a larger macro‐nutritional space: a greater variation in forage quality, among which they can select depending on their daily nutrient balancing requirements. While pine forests of different ages provide moose with a much higher abundance of ericaceous shrubs than spruce forests normally do (Hedwall et al., 2025), our results indicate that spruce forests hide some nutritionally well‐balanced shrub parcels under their canopy, at least when considering protein and digestible carbohydrates. It is also noteworthy that the bilberry–cowberry buffet available in spruce‐dominated stands provides the consumer with a larger variation in nutritional composition (larger nutritional space) than in pine‐dominated stands (Figure 4F).

Importantly, however, from an optimal foraging point of view (Belovsky, 1984), these parcels may be too sparsely distributed and in too low abundance to make it worthwhile for the large‐bodied moose to spend energy seeking them out, particularly if they need to share these resources with other cervids. Observations from southern Sweden suggest that due to feeding competition from smaller cervid species, moose can be replaced as consumers of the shrub layer (they eat less *Vaccinium*) and driven towards higher foraging strata that offer larger bites (they eat more Scots pine) (Spitzer et al., 2021).

### Caveats and future research recommendations

We focused on the winter season, when *Vaccinium* is a particularly important staple food for Swedish cervids if snow is not too deep (Spitzer, 2019). Winter is also the period when the majority of browsing damage is caused by moose to Scots pine production trees (Bergqvist et al., 2018), meaning that the quality of complementary food items such as *Vaccinium* shrubs is of particular relevance from a management perspective. However, there is of course variation in the intake of *Vaccinium* across seasons depending on the availability of other forage (e.g., Cederlund et al., 1980; Spitzer, 2019). While winter has long been seen as a nutritional bottleneck for cervids, a growing body of research suggests that summer nutrition is equally, if not more, important for their fitness (Cook et al., 2004; Schrempp et al., 2019). Because seasonality generally plays a large role in plant resource allocation (Chapin III et al., 1990), one may expect the *Vaccinium* shrubs to respond differently to canopy layer and soil conditions during the growing season compared to the dormant season, with relevant outcomes for herbivores.

Herbivores can themselves influence the chemical composition of the forage via the plant's chemical response to damage inflicted on their tissues (e.g., Persson et al., 2012; Schrijvers‐Gonlag et al., 2020; Stolter, 2008). A plant may in fact alter its chemical composition due to stimuli from the consumer, as has been shown for insects (Waterman et al., 2019) and sheep saliva (Liu et al., 2012). Relatedly, biomass loss to herbivory can affect next year's resource allocation in plants, as indicated by reduced growth and reproduction in bilberry due to high cervid browsing intensity (Hegland et al., 2005, 2010). In our sample collection, we avoided shoots with signs of recent ungulate or insect damage, but did not know if the sampled ramets had been browsed during previous years. We also did not include any estimate of local cervid densities in our data analyses, as available estimates would have been impossible to match with the plot level data of the shrubs, trees, and soil. We have, however, not found evidence to suggest that browsing by free‐ranging cervids significantly affects the annual shoot production of cowberry and bilberry in our study system (Juvany, 2023). Nevertheless, we cannot rule out that these plant species respond to browsing by changing their chemistry, and that such responses interact with the proxies we use for light availability (basal area and tree species composition). We suggest that conducting controlled browsing simulation studies is one way of assessing such effects. In addition, we recommend future research to incorporate seasonal aspects and inter‐annual differences in assessments of shrub nutritional composition to evaluate the consequences of varying forage qualities on the consumers, and that more types of secondary metabolites are included in assessments of overstory influences on the nutritional value of shrub forage.

### Implications for game and forest management

Previous results have highlighted the population‐level implications of intensively managed spruce forests for a variety of plant, fungi, and animal species (Felton, Hedwall, et al., 2021; Hedwall, Holmström, et al., 2019; Lindbladh et al., 2017; Strengbom et al., 2011). We can now add to these implications that dense spruce forests can also significantly alter the nutritional composition of important cervid forage species in the understory. Our results suggest that bilberry and cowberry plants that grow in spruce‐dominated forests may not only struggle with poor conditions for growth (Juvany, 2023) and reproduction (Eriksson & Fröborg, 1996) but may also be disproportionately vulnerable to browsing damage. This is because they may be more attractive as forage by moose, compared to their conspecifics growing in Scots pine stands (the same is likely to apply for smaller cervid species, but this has not been assessed). However, we also found that shrubs growing under dense spruce overstories had higher levels of lignin in their tissues, which may reduce their attractiveness as forage. It remains unclear how the combined changes to these two nutritional aspects affect the animals' browsing preferences, and the plants' resultant vulnerability to browsing. What also remains unknown is how global warming may alter the nutritional quality of forage from these plant species, either directly through physiological pathways (Lowman et al., 2022) or indirectly through forest owners' management decisions in response to climate change, which can readily alter tree species composition and density in production forests (Felton et al., 2024).

The difference in shrub nutritional composition observed could have implications for damage levels on Scots pine trees within browsing height. If moose consume *Vaccinium* forage found in dense spruce‐dominated forests, they may require less Scots pine to reach their preferred nutritional target (Figure 4). However, the chance that this will have a substantial ameliorating effect on Scots pine damage levels on a landscape scale is small, as any potential benefits are likely counteracted by the much lower availability of the shrubs' forage biomass in such environments (Juvany et al., 2023). Similarly, this lower availability likely also counteracts any potential effect that the different nutritional composition of shrub forage in these shaded environments has on the fitness of moose and other cervids. Regardless, our results highlight an additional beneficial aspect of diverse landscapes: even small differences in structure among production forests can increase the nutritional variation among natural forage plants for cervids. As such, our study adds to the pool of research suggesting large‐scale ecosystem and societal benefits from more varied forest landscapes (e.g., Brockerhoff et al., 2017; Felton et al., 2024). Potentially, this variation could also be created at the stand level. Some degree of thinning in a spruce‐dominated forest could result in a sweet spot whereby forage availability and variation in forage qualities are increased, without unacceptably reducing timber volumes at final felling, but further research is needed to test the effect of such management strategies.

In summary, our results demonstrate that changes in overstory conditions alter the nutritional composition of understory shrubs in ways that are of direct relevance to cervid foraging decisions in boreal forest systems. What these results highlight is that observations of long‐term changes in plant species cover and biomass should be accompanied by corresponding consideration of how associated drivers alter their nutritional composition.

## AUTHOR CONTRIBUTIONS

Annika M. Felton, Per‐Ola Hedwall, Adam Felton, and Anders Jarnemo formulated the idea. Annika M. Felton, Laura Juvany, Per‐Ola Hedwall, Julien Morel, and Alina Sayn developed the methodology. Laura Juvany, Annika M. Felton, Alina Sayn, Julien Morel, and Julia Erbrech conducted the field‐ and lab work. Laura Juvany, Alina Sayn, Julia Erbrech, and Julien Morel performed statistical analyses. Annika M. Felton, Laura Juvany, Per‐Ola Hedwall, Adam Felton, Julia Erbrech, Alina Sayn, Julien Morel, Märtha Wallgren, Anders Jarnemo, Leonie Schönbeck, and Robert Spitzer interpreted results and wrote the manuscript. Our study brings together authors from five different countries, including scientists based in Sweden where the study was carried out. Whenever relevant, literature published by scientists from the region has been cited, and efforts were made to include work published in the local language.

## CONFLICT OF INTEREST STATEMENT

The authors declare no conflicts of interest.

## Supporting information


Appendix S1.

Appendix S2.

Appendix S3.

## Data Availability

Data (Juvany, 2025) are available in Zenodo at https://doi.org/10.5281/zenodo.17734917.
