# Supplementary material for: Forest stand characteristics drive the macronutrient composition of Vaccinium winter forage for cervids
Source: Ecol Appl. 2026 Feb 5;36(1):e70182. doi: 10.1002/eap.70182 (PMC12874200; doi:10.1002/eap.70182)
Supplement: Supplementary file 1 — Appendix S1. [file EAP-36-e70182-s001.pdf]

Forest stand characteristics drive the macronutrient composition of *Vaccinium* winter forage for cervids

Annika M. Felton, Laura Juvany, Per-Ola Hedwall, Adam Felton, Julia Erbrech, Alina Sayn, Julien Morel,

Märtha Wallgren, Anders Jarnemo, Leonie Schönbeck and Robert Spitzer

APPENDIX S1: Supplementary Methods

Selecting stands based on site fertility estimates

Site fertility was estimated through available data on site index (SI) from each area. We obtained SI values for all available stands in each area and calculated their mean and median value, selecting a cut-off value close to both the mean and the median. Then, from all available stands in each site, we selected one stand with SI above the cut-off value (high fertility) and one below (low fertility), for each age category and tree species per area.

Plot measurements and calculations: tree layer, site index and soil samples

We measured the height of the tree with the largest DBH in each plot per stand and determined their age by coring at breast height (BH). If the largest tree had a crooked or damaged stem, and was therefore not suitable for aging, we selected the next largest tree. We calculated the total basal area per plot (m^2 ha^{-1}) as well as each tree species' contribution to it. We calculated our own value of stand site index (SI_c; tree height in m at 100 years´ age) for each stand following the equations in Elfving and Kiviste (1997). We used the height and total age obtained for the biggest trees in two plots, plus an additional plot using the same sampling methodology (used in an additional study), and calculated an average SI_c per stand using all three plots. We calculated the average of the age values measured from the tree cores. However, because age was measured from a core sample taken at BH, we estimated total age taking into account latitude and general fertility characteristics of each area, as in Hägglund and Lundmark (2003).

Within each circular plot, but outside the 2x2m centre square, we collected a total of four soil samples (Figure 1 main text). We removed the litter from the top and collected 10cm top soil including both humus and mineral soil fractions. We avoided rocks and other impediments. The four samples from the same plot were pooled together and dried at 60°C to remove most moisture. The total carbon (C) and nitrogen (N) content in the soil was determined through dry combustion according to ISO10694 (1995) and ISO

13878 (1998) respectively, using an elemental analyser for macrosamples (TruMac® CN, Leco corp, S:t Joseph, MI, USA). We calculated and used the carbon-to-nitrogen ratio (C:N) in the analysis. Nitrogen is the main nutrient that restricts productivity in our study systems, and the C:N ratio is a useful proxy for variation in soil nitrogen availability (Hedwall et al. 2019). We measured the pH using a one-part soil to 4 parts solution (1.47g $CaCl_2$ 1L H_2O). To extract a soil moisture value for each plot, we used data available through the SLU moisture map (SLU Markfuktighetskarta (Ågren and Lidberg 2020)).

Chemical analyses

Due to the high costs associated with wet chemistry analyses, we used near-infrared spectroscopy (NIRS) to estimate concentrations of the nutritional constituents, with a subset of representative samples also analysed using wet chemistry for calibration purposes (as per Vance et al. 2016). Each ground sample was thoroughly mixed before taking ca 10 g for scanning. NIRS reflectance spectra were acquired with a hyperspectral camera (Specim SWIR) from 935 to 2548 nm with 288 bands resulting in hypercubes with a spectral sampling of approximately 5.6 nm. A spectralon white target was used as a reference for converting the hyperspectral images to reflectance factor values. All 248 samples were scanned, with background removed and first and last ten bands discarded to reduce potential noise effects. After scanning, the most representative samples per plant species were selected for chemical analyses based on scores of principal component analysis (PCA) performed with the *prcomp* function from the “stats” R package. The first two principal components together represented 97.7% of the total explained variance. Samples were selected as representative (i.e. spanning the entire range of variation in concentrations of nutritional constituents) depending on their distance from the centre of the first (accounting for 84.5% of the total explained variance) and second (13.2%) principal components, which resulted in 87 samples selected for laboratory analyses. Specifically, 43 bilberry and 44 cowberry samples were selected (i.e. 35% of the whole population of samples).

The selected samples were sent to the laboratory DairyOne©, USA, for chemical analyses. Plant samples were analyzed for ash, total nitrogen, acid-detergent fiber (ADF), ADF-N (insoluble nitrogen within the ADF fraction), crude fat, amylase and sodium sulfite treated neutral-detergent fiber (aNDF), lignin, starch and water-soluble carbohydrates (WSC) using conventional wet chemistry techniques (Appendix S1 Supplementary methods). Results from the wet chemistry analyses of the representative subset of plant samples (n = 87) were used to adjust multivariate regression models where NIRS spectra were used as explanatory variables and above-mentioned laboratory-measured traits were used as response variables. The “caret” R package (Kuhn 2008) was used to adjust random forest regressions using a 10-

fold cross-validation and a grid search to optimize the parameters. Obtained models showed acceptable to good performances, with regression lines close to the 1:1 line; prediction errors (expressed as rRMSE) ranging between 1 and 11%; and with R^2 ranging between 0.91 and 0.94 (Appendix S2 Table S2), except for starch and ash that showed below acceptable performances (rRMSE of 30 and 16%, respectively). In the following data analyses, NIRS-predicted values were used for all samples and traits, except for starch and ash, where laboratory-obtained data were used after sending back all samples for wet chemistry analysis.

A subset of collected plant samples (see main text) were analyzed for total nitrogen, acid-detergent fiber (ADF), ADF-N (insoluble nitrogen within the ADF fraction), lipids, amylase and sodium sulfite treated neutral-detergent fiber (aNDF), lignin, starch, and water-soluble carbohydrates (WSC). All these parameters were analyzed using conventional wet chemistry techniques, at the Dairy One laboratory, Ithaca, USA. Ash (total minerals) was determined by ignition at 550 °C for 3 h. Nitrogen concentration (total N) was estimated by combustion using a CN628 Carbon/Nitrogen Determinator. Lignin and ADF were determined by using the ANKOM Technology method (Method 5 and 9 respectively), using solutions as in AOAC (1977). The ADF residue was analysed using a Leco TruMac N Macro Determinator to determine the protein fraction bound to the acid detergent fiber (ADF-N). aNDF was determined by using heat stable α-amylase and sodium sulphite, according to the ANKOM filter bag technique (ANKOM 2017), with solutions as in van Soest et al (1991). To estimate crude fat we applied ether extraction (AOAC 2003) using a Soxtec HT6 System with anhydrous diethyl ether. Crude fat residue was then determined gravimetrically after drying. We estimated water-soluble carbohydrates by incubating samples with water in a 40°C bath for 1 hour extracting simple sugars and fructan. Concentrations were then determined using a Thermo Scientific Genesys 10S Vis Spectrophotometer after acid hydrolysis with sulfuric acid and colorimetric reaction with potassium ferricyanide (Hall et al. 1999). We estimated starch concentrations enzymatically (glucoamylase) using an YSI 2700 Select Biochemistry Analyzer. All results were reported as % of true dry matter.

Outlier removals for data analyses

Some outliers were removed in relation to analyses that included data on stand characteristics. We removed one data point from a plot with a recorded C:N of 82.5, as all other results in the study were between 18-50, and we therefore deemed it a measurement error. We also removed four data points (two plots with bilberry and cowberry collected from each plot) where the stand index value was noted

as 12.5. This was much lower than all other stands in the study (19.5-37.9), and we deemed it to be a mistake.

Equations from the best fitting models to predict the ratio of AP:TCH for bilberry and cowberry.

Due to the large differences in the range of our predictors, we centred and standardized all independent variables, subtracting their mean and dividing by their standard deviation (SD). To predict AP:TCH from a given value of our predictors, this needs to be standardized first as follows:

Standardized Value = (Original Value – Mean) / SD

The mean and standard deviation from the original values of the variables used in our models.

	Mean		SD	
	Bilberry	Cowberry	Bilberry	Cowberry
Total basal area	23.53	23.26	12.88	12.55
Spruce percentage	30.34	27.90	38.41	37.41
Spruce percentage ^2	2383.06	NA	3659.72	NA
C:N	32.29	32.48	5.16	5.18

Due to the beta error distribution response of AP:TCH and logit link function for both bilberry and cowberry, we used the following formula:

$$\text{AP:TCH} = \frac{\exp(X)}{(1+\exp(X))}$$

Where X is the linear predictor, calculated as follows for bilberry:

X = Intercept + β1*Total basal area + β2*% Spruce + β3*(% Spruce^2) + β4*C:N + b1 + b2

And as follows for cowberry:

X = Intercept + β1*Total basal area + β2* % Spruce + β4*C:N + b1 + b2

Where Intercept is the model intercept, β1, β2, β3 and β4 are the coefficients estimated for each fixed effect and b1 and b2 correspond to the random fixed effects variances.

The intercepts and coefficients of the fixed effects, and variances for each random effect included in bilberry and cowberry best fitting models.

	Coefficient	
	Bilberry	Cowberry
Intercept	-2.30	-2.65
β1 (Total basal area)	0.04	0.10
β2 (Spruce percentage)	0.15	0.06
β3 (Spruce percentage ^2)	-0.14	NA

β4 (C:N)	-0.06	-0.04
b1 (Stand)	0.007	0.010
b2 (Site)	0.007	0.019

References

ANKOM. 2017. Neutral Detergent Fiber in Feeds - Filter Bag Technique (for A200 and A200I) Method 6. ANKOM Technology.

AOAC. 1977. Fiber (acid detergent) and lignin (H2SO4) in animal feed

Pages:2. Association of Official Agricultural Chemists.

AOAC. 2003. Crude Fat in Feeds,Cereal Grains,and Forages - RandaIl/Soxtec/Hexanes Extraction-Submersion Method. Association of Official Agricultural Chemists

Elfving, B., and A. Kiviste. 1997. Construction of site index equations for Pinus sylvestris L. using permanent plot data in Sweden. Forest Ecology and Management **98**:125-134.

Hall, M. B., W. H. Hoover, J. P. Jennings, and T. K. M. Webster. 1999. A method for partitioning neutral detergent-soluble carbohydrates. Journal of the Science of Food and Agriculture **79**:2079-2086.

Hedwall, P. O., E. Holmström, M. Lindbladh, and A. Felton. 2019. Concealed by darkness: How stand density can override the biodiversity benefits of mixed forests. Ecosphere **10**:e02835.

Hägglund, B., and J.-E. Lundmark. 2003. Handledning i bonitering med Skogshögskolans boniteringssystem. D. 2, Diagram och tabeller. Skogsstyr.

Kuhn, M. 2008. Building predictive models in R using the caret package. Journal of statistical software **28**:1-26.

Van Soest, P. J., J. B. Robertson, and B. A. Lewis. 1991. Methods for dietary fiber, neutral detergent fiber, and nonstarch polysaccharides in relation to animal nutrition. Journal of Dairy Science **74**:3583-3597.

Vance, C. K., D. R. Tolleson, K. Kinoshita, J. Rodriguez, and W. J. Foley. 2016. Near Infrared Spectroscopy in Wildlife and Biodiversity. Journal of Near Infrared Spectroscopy **24**:1-25.

Ågren, A., and W. Lidberg. 2020. Dokumentation nya hydrografiska kartor - vattendrag och SLU Markfuktighetskartor. Swedish University of Agricultural Sciences.
