# Supplementary material for: Forest stand characteristics drive the macronutrient composition of Vaccinium winter forage for cervids
Source: Ecol Appl. 2026 Feb 5;36(1):e70182. doi: 10.1002/eap.70182 (PMC12874200; doi:10.1002/eap.70182)
Supplement: Supplementary file 2 — Appendix S2. [file EAP-36-e70182-s003.pdf]

Ecological Applications

Forest stand characteristics drive the macronutrient composition of *Vaccinium* winter forage for cervids

Annika M. Felton, Laura Juvany, Per-Ola Hedwall, Adam Felton, Julia Erbrech, Alina Sayn, Julien Morel,

Märtha Wallgren, Anders Jarnemo, Leonie Schönbeck and Robert Spitzer

APPENDIX S2: Supplementary tables

Table S1. Data on the forest stands sampled in each of the five study areas (Figure 1 main text), including the age of the stand in years, age category from 1 to 4 (1= 7-18 years, 2= 19-39, 3= 40-70 and 4= >71 years old), its site index (SI_c), and the mean of total, Scots pine (*Pinus sylvestris*), Norway spruce (*Picea abies*) and all broadleaves basal area (BA, m^2/ha) calculated from all sampled plots in a stand.

Study area	Stand	Age (years)	Age Category	SI_c (meters)	Mean BA	Mean Pine BA	Mean Spruce BA	Mean Broadleaves BA
**S**	1	82	4	27	33.10	31.20	1.90	0.00
	2	40	2	30	33.83	31.04	2.68	0.11
	3	101	4	33	46.82	10.64	36.17	0.01
	4	53	3	25	32.32	30.31	1.61	0.39
	5	40	2	35	36.38	6.98	27.13	2.27
	6	10	1	36	6.65	4.81	0.07	1.77
	7	12	1	38	7.91	1.46	4.98	1.46
	8	15	1	34	18.56	17.38	0.00	1.17
	9	33	2	29	21.35	18.10	2.61	0.63
	10	60	3	31	27.44	24.87	2.53	0.04
	11	84	4	27	36.18	9.35	25.50	1.33
	12	14	1	32	21.60	0.00	20.86	0.74
	13	33	2	32	24.79	0.00	24.79	0.00
	14	94	4	22	26.29	25.46	0.48	0.35
**C**	1	75	4	27	38.40	2.81	35.43	0.17
	2	37	2	27	21.81	18.28	0.96	2.57
	3	31	2	22	19.48	19.47	0.00	0.00
	4	67	4	24	32.84	31.36	0.87	0.62
	5	63	3	28	34.99	34.62	0.05	0.32
	6	7	1	34	0.57	0.41	0.14	0.03
	7	9	1	38	0.57	0.01	0.56	0.00
	8	66	4	29	39.98	3.90	36.08	0.00
	9	49	3	33	37.58	0.00	37.24	0.34
	10	6	1	35	0.44	0.41	0.00	0.03

	11	10	1	37	0.73	0.00	0.73	0.00
	12	26	2	33	36.95	1.07	25.19	10.69
	13	27	2	29	21.55	10.19	11.33	0.03
	14	62	3	24	24.90	24.82	0.08	0.00
	15	152	4	13	18.59	18.59	0.00	0.00
	16	46	3	32	40.75	0.00	40.58	0.17
**N1**	1	68	4	22	29.54	29.53	0.01	0.00
	2	71	4	21	26.84	26.84	0.00	0.00
	3	85	4	28	54.42	16.47	37.95	0.00
	4	14	1	29	1.39	0.06	1.33	0.00
	5	51	3	24	24.04	21.54	2.51	0.00
	6	62	3	23	39.21	6.89	29.07	3.26
	7	26	2	25	14.85	3.95	9.60	1.30
	8	19	2	25	5.98	0.00	5.96	0.02
	9	50	3	23	16.81	1.66	15.11	0.04
	10	80	4	26	43.32	6.61	34.36	2.35
	11	31	2	26	27.54	24.94	1.05	1.55
	12	6	1	36	0.23	0.23	0.00	0.00
	13	8	1	32	0.70	0.65	0.00	0.05
	14	16	2	23	16.11	14.52	0.05	1.54
	15	44	3	24	33.55	21.97	8.53	3.05
**SE**	1	75	4	31	27.52	23.95	3.57	0.00
	2	76	4	30	33.74	25.89	7.85	0.00
	3	57	3	28	26.70	26.05	0.30	0.35
	4	29	2	33	16.22	15.79	0.43	0.00
	5	42	3	31	24.25	23.68	0.48	0.09
	6	NA		NA	18.54	18.54	0.00	0.00
	7	63	3	28	23.95	21.76	1.37	0.82
	8	60	3	30	32.78	32.69	0.09	0.00
	9	55	3	30	26.52	22.90	3.60	0.02
	10	57	3	25	22.57	19.37	2.11	1.09
**N2**	1	60	3	27	23.17	23.17	0.00	0.00
	2	45	3	29	27.55	26.44	0.37	0.74
	3	51	3	30	33.01	29.87	3.14	0.00
	4	51	3	25	17.37	13.77	3.01	0.59
	5	82	4	20	17.16	16.50	0.65	0.00
	6	55	3	27	18.64	18.33	0.28	0.02
	7	60	3	27	29.86	29.86	0.00	0.00
	8	54	3	27	18.02	17.22	0.73	0.06
	9	62	3	25	16.56	13.66	2.90	0.00
	10	41	3	28	21.83	21.83	0.00	0.00

Table S2. Summary statistics of the NIRS models for the variables acid detergent fiber (ADF), ADF-N (insoluble nitrogen within the ADF fraction), ash, crude fat (lipids), lignin, total nitrogen, amylase and sodium sulfite treated neutral-detergent fiber (aNDF), starch and water soluble carbohydrates (WSC); including information regarding model R^2, RMSE (root-mean-square error), rRMSE (prediction error as % of dry matter), slope and intercept.

Trait of interest	R^2	RMSE	rRMSE (%)	Slope	Intercept
**ADF**	0.93	1.55	4.0	1.14	-5.30
**ADF-N**	0.92	0.26	6.7	1.28	-1.09
**Ash**	0.8	0.51	15.9	1.35	-1.15
**Crude fat**	0.92	0.36	8.5	1.15	-0.63
**Lignin**	0.94	0.93	4.2	1.12	-2.69
**Total nitrogen**	0.91	0.06	5.1	1.22	-0.25
**aNDF**	0.91	1.71	3.6	1.18	-8.43
**Starch**	0.85	0.44	29.7	2.04	-1.55
**WSC**	0.91	2.24	10.8	1.14	-3.00

Table S3. Average concentrations (g 100g^{-1}) of seven macro-nutritional parameters and energy content (kJ g^{-1}) of bilberry (BB) and cowberry (CB) browse collected winter time in five study areas. Averages are listed per study area, and overall averages (Total) and SE per species, with minimum and maximum observations. Statistical results from linear mixed-effects models (LMM) display differences between the two plant species per parameter.

**Site**	**Lignin**		**AP**		**TNC**		**Ash**		**Crude Fat**		**Cellulose**		**Hemicellulose**		**Energy**	
	BB	CB	BB	CB	BB	CB	BB	CB	BB	CB	BB	CB	BB	CB	BB	CB
**S**	26.5	21.7	4.3	4.0	14.5	23.5	3.8	2.8	3.9	5.1	18.4	14.9	8.5	8.6	9.1	10.5
**N2**	23.7	18.8	3.6	2.9	17.4	29.1	4.3	3.2	3.9	5.2	17.6	15.2	7.8	8.4	9.2	11.2
**SE**	25.0	20.2	4.4	3.6	15.4	26.0	3.8	3.3	4.0	5.3	18.6	15.3	8.8	8.4	9.4	10.9
**C**	25.2	19.7	3.5	3.1	16.4	27.7	3.7	3.2	2.9	4.7	19.6	15.6	7.4	8.6	8.9	11.0
**N1**	24.8	19.3	3.9	3.0	16.2	26.6	3.5	2.8	3.0	4.8	18.8	15.0	7.6	8.3	8.9	10.7
**Total**	**25.1**	**19.9**	**3.9**	**3.3**	**16.0**	**26.6**	**3.8**	**3.0**	**3.5**	**5.0**	**18.7**	**15.2**	**8.0**	**8.5**	**9.1**	**10.8**
**SE**	**1.6**	**1.8**	**0.6**	**0.8**	**2.1**	**3.5**	**0.9**	**0.8**	**0.6**	**0.5**	**1.1**	**0.7**	**0.7**	**0.6**	**0.3**	**0.6**
**Min**	21.6	16.9	2.4	1.1	11.9	21.2	2.2	1.5	2.2	3.9	16.2	12.7	6.5	6.9	7.9	9.7
**Max**	29.0	24.0	5.8	4.5	20.4	36.0	6.8	7.2	4.5	5.9	21.8	16.8	9.4	10.7	9.9	12.3
**Coefficient**	25.1	-5.2	3.9	-0.6	15.9	10.6	3.8	-0.8	3.5	1.6	18.7	-3.5	7.9	0.5	907.5	174.6
**SE**	0.2	0.2	0.1	0.1	0.3	0.2	0.1	0.1	0.1	0.1	0.1	0.1	0.1	0.1	4.33	5.4
**z-statistic**	162.4	-33.8	64.0	-9.5	62.5	45.4	49.0	-7.3	71.7	30.2	222.9	-34.5	139.1	6.1	209.5	32.4
**p-value**	<0.001		<0.001		<0.001		<0.001		<0.001		<0.001		<0.001		<0.001	

Table S4. Principle component coefficients of the macro-nutritional composition of bilberry and cowberry samples collected wintertime in five locations across Sweden (Figure 1). For each plant sample, the concentration (g 100 g^{-1} dm) of available protein (AP), ash, crude fat, total non-structural carbohydrates (TNC), cellulose, hemicellulose and lignin are included. Values > 0.7 and values < -0.7 on PC1 and PC2 are indicated in bold, as these were interpreted as being those variables (also bold) that contributed most strongly to explaining the variation along the respective axis.

	**Bilberry**			**Cowberry**		
	PC1	PC2	PC3	PC1	PC2	PC3
**AP**	**0.66**	0.35	0.01	**0.80**	-0.23	0.39
**Ash**	-0.21	0.28	0.90	-0.17	0.33	0.49
**Crude Fat**	-0.06	**0.92**	-0.04	-0.36	**-0.65**	0.46
**Cellulose**	0.61	-0.58	0.02	0.03	**0.85**	-0.12
Hemicellulose	0.54	0.59	-0.33	0.41	0.53	0.55
**Lignin**	**0.87**	-0.11	0.21	**0.90**	-0.17	-0.08
**TNC**	**-0.93**	-0.01	-0.18	**-0.88**	0.10	0.24
**% Variance**	39.5	25.1	14.3	36.5	23.1	14.1

Table S5. Results from assessment (LMM) of the relationship between stand characteristics (centred and standardized variables) and the principle components (PC1 and PC2) in Figure 2 which is a measure of the macro-nutritional balance of the bilberry browse. For each PC, the table shows results from the full model results and also the best candidate models (see Methods). BA = basal area.

PC1 Bilberry		Total BA	%Spruce	Total BA X %Spruce	Soil C:N	Soil pH	Soil moisture	Site Index (SI_c)	Marg. R^2 / Cond. R^2	AIC	Delta AIC	Model weights
	Coefficient	0.76	0.27	0.16	-0.41	-0.17	-0.11	0.03				
**Full Model**	*SE*	0.14	0.12	0.10	0.17	0.12	0.11	0.16	0.45 /	352.5	3.3	0.19
	z-statistic	5.57	2.22	1.63	-2.42	-1.46	-1.03	0.16	0.80			
	P-Value	**<0.001**	**0.026**	0.103	**0.015**	0.145	0.303	0.874				
	Coefficient	0.73	0.30	0.14	-0.27							
**Selected**	*SE*	0.12	0.12	0.09	0.14				0.39 /	349.2	0	1.00
**Model**	*z-statistic*	6.07	2.50	1.54	-2.03				0.80			
	P-Value	**<0.001**	**0.012**	0.123	**0.042**							
	Coefficient	0.81	0.27		-0.30							
**Candidate**	*SE*	0.11	0.12		0.14				0.38 /	349.8	0.6	0.74
**Model 1**	*z-statistic*	7.33	2.27		-2.23				0.80			
	P-Value	**<0.001**	**0.023**		**0.026**							
	Coefficient	0.73	0.28	0.15	-0.38	-0.15						
**Candidate**	*SE*	0.12	0.12	0.09	0.16	0.12			0.42 /	349.5	0.3	0.86
**Model 2**	*z-statistic*	6.12	2.37	1.66	-2.41	-1.28			0.80			
	P-Value	**<0.001**	**0.018**	0.097	**0.016**	0.199						
	Coefficient	0.81	0.26		-0.39	-0.13						
**Candidate**	*SE*	0.11	0.12		0.16	0.12			0.41 /	350.2	1	0.61
**Model 3**	*z-statistic*	7.46	2.14		-2.50	-1.13			0.79			
	P-Value	**<0.001**	**0.033**		**0.012**	0.258						
	Coefficient	0.75	0.28	0.16	-0.42	-0.18	-0.11					
**Candidate**	*SE*	0.12	0.12	0.09	0.16	0.12	0.11		0.45 /	350.5	1.3	0.52
**Model 4**	*z-statistic*	6.18	2.32	1.69	-2.61	-1.48	-1.03		0.80			
	P-Value	**<0.001**	**0.020**	0.090	**0.009**	0.142	0.305					
	Coefficient	0.75	0.30	0.15	-0.30		-0.08					
**Candidate**	*SE*	0.12	0.12	0.09	0.14		0.11		0.41 /	350.6	1.4	0.50
**Model 5**	*z-statistic*	6.07	2.48	1.55	-2.14		-0.75		0.80			
	P-Value	**<0.001**	**0.013**	0.121	**0.032**		0.456					
	Coefficient	0.82	0.27		-0.32		-0.08			351.0	1.8	0.41

**Candidate Model 6**	*SE*	0.11	0.12		0.14		0.11		0.40 / 0.80			
	z-statistic	7.27	2.25		-2.34		-0.73					
	P-Value	**<0.001**	**0.025**		**0.019**		0.467					
**Candidate Model 7**	*Coefficient*	0.74	0.29	0.14	-0.26			0.03	0.40 / 0.80	351.1	1.9	0.39
	SE	0.14	0.12	0.10	0.14			0.16				
	z-statistic	5.46	2.38	1.48	-1.84			0.19				
	P-Value	**<0.001**	**0.018**	0.140	0.067			0.849				
**PC2 Bilberry**		**Total BA**	**%Spruce**	**Total BA X %Spruce**	**Soil C:N**	**Soil pH**	**Soil moisture**	**Site Index** (SI_c)	**Marg. R^2 / Cond. R^2**	**AIC**	**Delta AIC**	**Model weights**
**Full Model**	*Coefficient*	-0.08	0.05	-0.08	0.01	0.14	0.04	0.07	0.03 / 0.87	254.2	6.4	0.04
	SE	0.08	0.07	0.06	0.11	0.08	0.07	0.10				
	z-statistic	-0.93	0.64	-1.39	0.10	1.82	0.56	0.69				
	P-Value	0.352	0.523	0.165	0.923	0.069	0.575	0.493				
**Selected Model**	*Coefficient*	-0.13				0.13			0.02 / 0.86	247.8	0	1.00
	SE	0.06				0.06						
	z-statistic	-2.09				2.01						
	P-Value	**0.037**				**0.044**						
**Candidate Model 1**	*Coefficient*	-0.13	0.08			0.13			0.02 / 0.87	248.5	0.7	0.70
	SE	0.06	0.08			0.06						
	z-statistic	-2.21	1.23			2.03						
	P-Value	**0.027**	0.260			**0.043**						
**Candidate Model 2**	*Coefficient*	-0.09	0.06	-0.68		0.14			0.02 / 0.87	249.1	1.3	0.52
	SE	0.07	0.07	0.06		0.06						
	z-statistic	-1.37	0.94	-1.22		2.18						
	P-Value	0.170	0.345	0.224		**0.029**						
**Candidate Model 3**	*Coefficient*	-0.11				0.12		0.07	0.02 / 0.86	249.2	1.4	0.50
	SE	0.07				0.07		0.08				
	z-statistic	-1.62				1.86		0.78				
	P-Value	0.106				0.063		0.437				
**Candidate Model 4**	*Coefficient*	-0.14				0.13	0.04		0.02 / 0.86	249.5	1.7	0.43
	SE	0.06				0.06	0.07					
	z-statistic	-2.16				2.07	0.56					
	P-Value	**0.031**				**0.041**	0.579					
**Candidate Model 5**	*Coefficient*	-0.13			-0.05	0.11			0.02 / 0.86	249.5	1.7	0.43
	SE	0.06			0.10	0.08						

	z-statistic	-2.12	-0.49	1.46					
	P-Value	**0.034**	0.623	0.143					
**Candidate Model 6**	*Coefficient*	-0.13	-0.12			0.01 / 0.85	249.6	1.8	0.41
	SE	0.06	0.08						
	z-statistic	-2.04	-1.45						
	P-Value	**0.042**	0.147						
**Candidate Model 7**	*Coefficient*			0.11	0.12	0.01 / 0.85	249.7	1.9	0.39
	SE			0.07	0.08				
	z-statistic			1.63	1.51				
	P-Value			0.102	0.132				
**Candidate Model 8**	*Coefficient*	-0.12				0.01 / 0.85	249.7	1.9	0.39
	SE	0.06							
	z-statistic	-1.89							
	P-Value	0.059							

Table S6. Results from assessment (LMM) of the relationship between stand characteristics (centred and standardized variables) and the principle components (PC1 and PC2) in Figure 2 which is a measure of the macro-nutritional balance of the cowberry browse. For each PC, the table shows results from the full model results and also the best candidate models (see Methods). BA = basal area.

PC1 Cowberry		Total BA	%Spruce	Total BA X: %Spruce	Soil C:N	Soil pH	Soil moisture	Site Index (SI_c)	Marg.l R^2 / Cond. R^2	AIC	Delta AIC	Model weights
**Full Model**	*Coefficient*	0.63	0.53	0.23	-0.14	0.10	-0.03	0.03	0.45 / 0.66	360.9	5.1	0.08
	SE	0.13	0.11	0.08	0.18	0.13	0.11	0.15				
	z-statistic	4.99	4.85	2.75	-0.77	0.80	-0.27	0.21				
	P-Value	**<0.001**	**<0.001**	**0.006**	0.440	0.425	0.786	0.838				
**Selected Model**	*Coefficient*	0.61	0.52	0.23	-0.21				0.43 / 0.65	355.8	0	1.00
	SE	0.10	0.10	0.08	0.13							
	z-statistic	5.87	4.94	2.77	-1.58							
	P-Value	**<0.001**	**<0.001**	**0.006**	0.115							
**Candidate Model 1**	*Coefficient*	0.61	0.57	0.25		0.16			0.41 / 0.67	355.8	0	1.00
	SE	0.10	0.10	0.08		0.10						
	z-statistic	5.91	5.74	3.05		1.58						
	P-Value	**<0.001**	**<0.001**	**0.002**		0.113						
**Candidate Model 2**	*Coefficient*	0.60	0.57	0.25					0.41 / 0.65	356.3	0.5	0.78
	SE	0.10	0.10	0.08								
	z-statistic	5.80	5.65	3.09								
	P-Value	**<0.001**	**<0.001**	**0.002**								
**Candidate Model 3**	*Coefficient*	0.61	0.53	0.23	-0.14	0.11			0.42 / 0.66	357.0	1.2	0.55
	SE	0.10	0.11	0.08	0.16	0.12						
	z-statistic	5.92	5.05	2.83	-0.88	0.88						
	P-Value	**<0.001**	**<0.001**	**0.005**	0.378	0.378						
**Candidate Model 4**	*Coefficient*	0.64	0.55	0.24		0.16		0.07	0.41 / 0.66	357.5	1.7	0.43
	SE	0.12	0.11	0.08		0.10		0.13				
	z-statistic	5.35	5.16	2.82		1.50		0.53				
	P-Value	**<0.001**	**<0.001**	**0.005**		0.134		0.595				
**Candidate Model 5**	*Coefficient*	0.62	0.52	0.23	-0.22	-0.05			0.44 / 0.64	357.6	1.8	0.41
	SE	0.11	0.10	0.08	0.14	0.11						
	z-statistic	5.80	4.95	2.79	-1.63	-0.48						
	P-Value	**<0.001**	**<0.001**	**0.005**	0.103	0.629						
	Coefficient	0.65	0.54	0.24				0.10	0.42 / 0.64	357.7	1.9	0.39

**Candidate Model 6**	*SE*	0.12	0.11	0.08				0.13				
	z-statistic	5.37	5.02	2.81				0.73				
	P-Value	**<0.001**	**<0.001**	**0.005**				0.465				
**Candidate Model 7**	*Coefficient*	0.61	0.51	0.23	-0.21			0.01	0.43 / 0.65	357.8	2.0	0.37
	SE	0.12	0.11	0.08	0.15			0.15				
	z-statistic	5.00	4.76	2.70	-1.40			0.10				
	P-Value	**<0.001**	**<0.001**	**0.007**	0.161			0.919				
**PC2 Cowberry**		**Total BA**	**%Spruce**	**Total BA X: %Spruce**	**Soil C:N**	**Soil pH**	**Soil moisture**	**Site Index** (SI_c)	**Marg.l R^2 / Cond. R^2**	**AIC**	**Delta AIC**	**Model weights**
**Full Model**	*Coefficient*	0.26	0.35	-0.10	0.05	0.16	0.13	-0.33	0.23 / 0.50	367.1	4.6	0.10
	SE	1.14	0.12	0.09	0.18	0.13	0.11	0.16				
	z-statistic	1.90	2.93	-1.03	0.29	1.27	1.17	-2.05				
	P-Value	0.057	**0.003**	0.301	0.772	0.206	0.241	**0.041**				
**Selected Model**	*Coefficient*	0.22	0.36					-0.36	0.19 / 0.52	362.5	0	1.00
	SE	0.12	0.12					0.14				
	z-statistic	1.91	3.09					-2.47				
	P-Value	0.056	**0.002**					0.013				
**Candidate Model 1**	*Coefficient*	0.22	0.37			0.12		-0.37	0.21 / 0.52	363.3	0.8	0.67
	SE	0.11	0.12			0.11		0.14				
	z-statistic	1.90	3.16			1.12		-2.58				
	P-Value	0.058	**0.002**			0.263		0.010				
**Candidate Model 2**	*Coefficient*	0.19	0.36				0.10	-0.37	0.20 / 0.51	363.7	1.2	0.55
	SE	0.12	0.12				0.11	0.14				
	z-statistic	1.57	3.08				0.91	-2.55				
	P-Value	0.116	**0.002**				0.362	**0.011**				
**Candidate Model 3**	*Coefficient*	0.28	0.34	-0.08				-0.08	0.20 / 0.51	363.9	1.4	0.50
	SE	0.14	0.12	0.10				0.10				
	z-statistic	2.07	2.90	-0.82				-0.82				
	P-Value	**0.038**	**0.004**	0.411				0.411				
**Candidate Model 4**	*Coefficient*		0.41					-0.47	0.17 / 0.54	364.0	1.5	0.47
	SE		0.12					0.14				
	z-statistic		3.45					-3.42				
	P-Value		**<0.001**					**<0.001**				
**Candidate Model 5**	*Coefficient*		0.40				0.15	-0.46	0.20 / 0.54	364.1	1.6	0.45
	SE		0.12				0.11	0.13				

	z-statistic		3.37				1.39	-3.41				
	P-Value		**<0.001**				0.166	**<0.001**				
**Candidate Model 6**	*Coefficient*	0.18	0.37			0.13	0.11	-0.38	0.22 / 0.52	364.3	1.8	0.41
	SE	0.12	0.12			0.11	0.11	0.14				
	z-statistic	1.53	3.16			1.20	1.01	-2.67				
	P-Value	0.125	**0.002**			0.229	0.311	**0.008**				
**Candidate Model 7**	*Coefficient*	0.21	0.36		-0.07			-0.38	0.20 / 0.52	364.4	1.9	0.39
	SE	0.12	0.12		0.16			0.16				
	z-statistic	1.73	3.00		-0.42			-2.42				
	P-Value	0.083	**0.003**		0.671			**0.016**				
**Candidate Model 8**	*Coefficient*	0.28	0.35	-0.09		0.13		-0.34	0.21 / 0.51	364.5	2	0.37
	SE	0.13	0.12	0.09		0.11		0.14				
	z-statistic	2.11	2.96	-0.91		1.18		-2.34				
	P-Value	**0.035**	**0.003**	0.363		0.236		**0.019**				

Table S7. Results from assessment (GLMM) of the relationship between stand characteristics (centred and standardized variables) and the ratio between available protein (AP) and Total Carbohydrates (TCH = Total non-structural carbohydrates (TNC) + cellulose + hemicellulose) in bilberry and cowberry. The table shows results from the full model results and also the best candidate models (see Methods). BA = basal area.

**Bilberry AP:TCH**		**Intercept**	**Total BA**	**%Spruce**	**%Spruce^2**	**Total BA X %Spruce**	**Total BA X %Spruce^2**	**Soil C:N**	**Soil pH**	**Soil moisture**	**Site Index (SI_c)**	**Marg. R^2 / Cond. R^2**	**AIC**	**Delta AIC**	**Model weights**
**Full Model**	*Coefficient*	-2.30	0.04	0.16	-0.14	-0.01	0.01	-0.06	-0.00	-0.00	0.00	0.25 / 0.76	-727.8	9.6	0.01
	SE	0.04	0.02	0.06	0.06	0.05	0.05	0.02	0.02	0.01	0.02				
	z-statistic	-56.95	2.17	2.58	-2.43	-0.18	0.31	-2.57	-0.01	-0.29	0.08				
	P-Value	**<0.001**	**0.030**	**0.010**	**0.015**	0.859	0.760	**0.010**	0.989	0.775	0.939				
**Selected Model**	*Coefficient*	-2.30	0.04	0.15	-0.14			-0.06				0.24 / 0.76	-737.4	0	1.00
	SE	0.04	0.01	0.06	0.06			0.02							
	z-statistic	-56.18	2.86	2.57	-2.41			-3.40							
	P-Value	**<0.001**	**0.004**	**0.010**	**0.016**			**0.001**							
**Candidate Model 1**	*Coefficient*	-2.30	0.04	0.15	-0.14		0.01	-0.06				0.24 / 0.76	-735.7	1.7	0.43
	SE	0.04	0.02	0.06	0.06		0.01	0.02							
	z-statistic	-55.72	2.48	2.61	-2.43		0.55	-3.28							
	P-Value	**<0.001**	**0.013**	**0.009**	**0.015**		0.582	**0.001**							
**Candidate Model 2**	*Coefficient*	-2.30	0.04	0.15	-0.14	0.01		-0.06				0.24 / 0.76	-735.6	1.8	0.41
	SE	0.04	0.02	0.06	0.06	0.01		0.02							
	z-statistic	-55.80	2.42	2.59	-2.41	0.47		-3.28							
	P-Value	**<0.001**	**0.015**	**0.009**	**0.016**	0.636		**0.001**							
**Candidate Model 3**	*Coefficient*	-2.30	0.04	0.15	-0.14			-0.06		-0.00		0.25 / 0.76	-735.5	1.9	0.39
	SE	0.04	0.01	0.06	0.06			0.02		0.01					
	z-statistic	-57.63	2.86	2.59	-2.43			-3.38		-0.35					
	P-Value	**<0.001**	**0.004**	**0.010**	**0.015**			**0.001**		0.727					
**Candidate Model 4**	*Coefficient*	-2.30	0.04	0.15	-0.14			-0.06			0.00	0.24 / 0.76	-735.4	2	0.37
	SE	0.04	0.02	0.06	0.06			0.02			0.02				
	z-statistic	-56.29	2.59	2.53	-2.39			-3.10			0.09				
	P-Value	**<0.001**	**0.009**	**0.011**	**0.017**			**0.002**			0.932				
**Candidate Model 5**	*Coefficient*	-2.30	0.04	0.15	-0.14			-0.06	0.00			0.24 / 0.76	-735.4	2	0.37
	SE	0.04	0.01	0.06	0.06			0.02	0.02						
	z-statistic	-55.99	2.85	2.56	-2.40			-2.87	0.07						
	P-Value	**<0.001**	**0.004**	**0.010**	**0.016**			**0.004**	0.945						

Cowberry AP:TCH		Intercept	Total BA	%Spruce	%Spruce^2	Total BA X %Spruce	Total BA X %Spruce^2	Soil C:N	Soil pH	Soil moisture	Site Index (SI_c)	Marg. R^2 / Cond. R^2	AIC	Delta AIC	Model weights
**Full Model**	*Coefficient*	-2.61	0.09	0.12	0.00	-0.01	0.00	-0.06	-0.03	-0.01	0.02	0.28 / 0.55	-655.2	9.5	0.01
	SE	0.08	0.05	0.10	0.00	0.09	0.00	0.04	0.03	0.03	0.04				
	z-statistic	-30.10	1.64	1.21	-0.71	-0.06	0.27	-1.33	-0.83	-0.44	0.53				
	P-Value	**<0.001**	0.102	0.226	0.477	0.949	0.788	0.184	0.405	0.659	0.593				
**Selected Model**	*Coefficient*	-2.65	0.10	0.06				-0.05				0.24 / 0.60	-664.7	0	1.00
	SE	0.07	0.02	0.02				0.03							
	z-statistic	-39.75	4.78	2.41				-1.58							
	P-Value	**<0.001**	**<0.001**	**0.016**				0.113							
**Candidate Model 1**	*Coefficient*	-2.65	0.11	0.07								0.22 / 0.60	-664.2	0.5	0.78
	SE	0.07	0.02	0.02											
	z-statistic	-36.97	4.91	2.99											
	P-Value	**<0.001**	**<0.001**	**0.003**											
**Candidate Model 2**	*Coefficient*	-2.65	0.11								0.04	0.24 / 0.58	-664.0	0.7	0.70
	SE	0.07	0.02								0.03				
	z-statistic	-40.53	5.07								1.33				
	P-Value	**<0.001**	**<0.001**								0.182				
**Candidate Model 3**	*Coefficient*	-2.65	0.09	0.07		0.02						0.22 / 0.60	-663.6	1.1	0.58
	SE	0.07	0.03	0.02		0.02									
	z-statistic	-36.37	3.58	3.11		1.23									
	P-Value	**<0.001**	**<0.001**	**0.002**		0.220									
**Candidate Model 4**	*Coefficient*	-2.69	0.11		0.00			-0.05				0.23 / 0.60	-663.6	1.1	0.58
	SE	0.07	0.02		0.00			0.03							
	z-statistic	-39.33	4.89		2.16			-1.63							
	P-Value	**<0.001**	**<0.001**		**0.031**			0.102							
**Candidate Model 5**	*Coefficient*	-2.65	0.09	0.06		0.02		-0.04				0.24 / 0.60	-663.6	1.1	0.58
	SE	0.07	0.03	0.02		0.02		0.03							
	z-statistic	-38.90	3.63	2.53		0.94		-1.38							
	P-Value	**<0.001**	**<0.001**	**0.011**		0.347		0.169							
**Candidate Model 6**	*Coefficient*	-2.65	0.10	0.06				-0.06	-0.02			0.26 / 0.58	-663.4	1.3	0.52
	SE	0.06	0.02	0.02				0.04	0.03						
	z-statistic	-42.93	4.79	2.22				-1.77	-0.82						
	P-Value	**<0.001**	**<0.001**	**0.027**				0.076	0.415						
	Coefficient	-2.61	0.10	0.13	0.00			-0.05					-663.2	1.5	0.47

**Candidate Model 7**	*SE*	0.09	0.02	0.10	0.00		0.03			0.24 / 0.59			
	z-statistic	-28.84	4.53	1.26	-0.71		-1.62						
	P-Value	**<0.001**	**<0.001**	0.206	0.477		0.105						
**Candidate Model 8**	*Coefficient*	-2.65	0.11	0.05			-0.04		0.02	0.25 / 0.59	-663.2	1.5	0.47
	SE	0.06	0.02	0.03			0.04		0.04				
	z-statistic	-41.37	4.53	2.16			-1.11		0.70				
	P-Value	**<0.001**	**<0.001**	**0.031**			0.268		0.482				
**Candidate Model 9**	*Coefficient*	-2.68	0.12		0.00				0.05	0.23 / 0.58	-663.0	1.7	0.43
	SE	0.07	0.02		0.00				0.03				
	z-statistic	-40.15	5.27		2.13				1.44				
	P-Value	**<0.001**	**<0.001**		**0.033**				0.151				
**Candidate Model 10**	*Coefficient*	-2.69	0.11		0.00					0.21 / 0.61	-663.0	1.7	0.43
	SE	0.07	0.02		0.00								
	z-statistic	-36.57	5.05		2.75								
	P-Value	**<0.001**	**<0.001**		**0.006**								
**Candidate Model 11**	*Coefficient*	-2.65	0.10	0.06		0.02			0.03	0.23 / 0.58	-662.8	1.9	0.39
	SE	0.07	0.03	0.02		0.02			0.03				
	z-statistic	-39.32	3.68	2.49		0.93			1.07				
	P-Value	**<0.001**	**<0.001**	**0.013**		0.352			0.282				
**Candidate Model 12**	*Coefficient*	-2.65	0.10	0.06			-0.05	-0.00		0.24 / 0.60	-662.7		
	SE	0.07	0.02	0.02			0.03	0.02				2	0.37
	z-statistic	-40.02	4.54	2.41			-1.57	-0.09					
	P-Value	**<0.001**	**<0.001**	**0.016**			0.117	0.932					

References

SMHI. 2023. Swedish Meteorological and Hydrological Institute, Arkiverade kartor för normalperioden 1991-2020.
