# Supplementary material for: Forest stand characteristics drive the macronutrient composition of Vaccinium winter forage for cervids
Source: Ecol Appl. 2026 Feb 5;36(1):e70182. doi: 10.1002/eap.70182 (PMC12874200; doi:10.1002/eap.70182)
Supplement: Supplementary file 3 — Appendix S3. [file EAP-36-e70182-s002.pdf]

Ecological Applications

Forest stand characteristics drive the macronutrient composition of *Vaccinium* winter forage for cervids

Annika M. Felton, Laura Juvany, Per-Ola Hedwall, Adam Felton, Julia Erbrech, Alina Sayn, Julien Morel,

Märtha Wallgren, Anders Jarnemo, Leonie Schönbeck and Robert Spitzer

Appendix S3. Supplementary figures

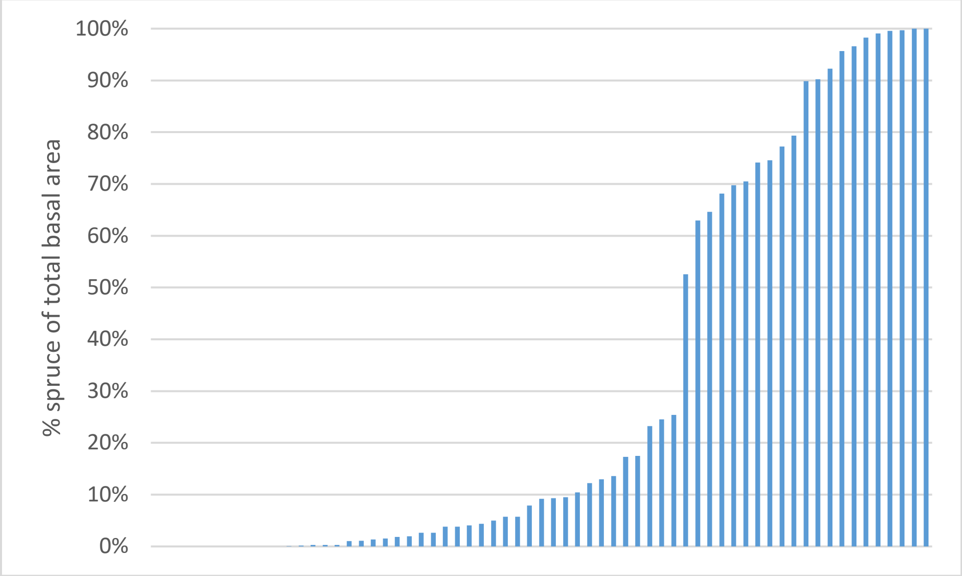

Figure S1. Percentage spruce of total basal area per stand sampled in the study (n = 65 stands, on x-axis). Data shows mean of two plots per stand.
